# Biodiversity of Duckweed (Lemnaceae) in Water Reservoirs of Ukraine and China Assessed by Chloroplast DNA Barcoding

**DOI:** 10.3390/plants11111468

**Published:** 2022-05-30

**Authors:** Guimin Chen, Anton Stepanenko, Olha Lakhneko, Yuzhen Zhou, Olena Kishchenko, Anton Peterson, Dandan Cui, Haotian Zhu, Jianming Xu, Bogdan Morgun, Dmitri Gudkov, Nikolai Friesen, Mykola Borysyuk

**Affiliations:** 1 Jiangsu Key Laboratory for Eco-Agricultural Biotechnology around Hongze Lake, School of Life Sciences, Huaiyin Normal University, Huai’an 223300, China; cgm@hytc.edu.cn (G.C.); stepanenko@hytc.edu.cn (A.S.); zyz@hytc.edu.cn (Y.Z.); o_kishchenko@hotmail.com (O.K.); peterson@hytc.edu.cn (A.P.); cuidandan.meizi@foxmail.com (D.C.); haotianzhu567@163.com (H.Z.); xjm@hytc.edu.cn (J.X.); 2 Institute of Cell Biology and Genetic Engineering, National Academy of Sciences of Ukraine, 03143 Kyiv, Ukraine; olakhneko@icbge.org.ua (O.L.); bmorgun@icbge.org.ua (B.M.); 3 Institute of Hydrobiology, National Academy of Sciences of Ukraine, 04210 Kyiv, Ukraine; digudkov@gmail.com; 4 Botanical Garden of the University of Osnabrück, 49074 Osnabrück, Germany; nfriesen@uni-osnabrueck.de

**Keywords:** aquatic plants, duckweed, biodiversity, barcoding, chloroplast DNA, molecular evolution

## Abstract

Monitoring and characterizing species biodiversity is essential for germplasm preservation, academic studies, and various practical applications. Duckweeds represent a group of tiny aquatic plants that include 36 species divided into 5 genera within the Lemnaceae family. They are an important part of aquatic ecosystems worldwide, often covering large portions of the water reservoirs they inhabit, and have many potential applications, including in bioremediation, biofuels, and biomanufacturing. Here, we evaluated the biodiversity of duckweeds in Ukraine and Eastern China by characterizing specimens using the two-barcode protocol with the chloroplast *atpH–atpF* and *psbK–psbI* spacer sequences. In total, 69 Chinese and Ukrainian duckweed specimens were sequenced. The sequences were compared against sequences in the NCBI database using BLAST. We identified six species from China (*Spirodela polyrhiza*, *Landoltia punctata*, *Lemna aequinoctialis*, *Lemna minor*, *Lemna turionifera*, and *Wolffia globosa*) and six from Ukraine (*S. polyrhiza*, *Lemna gibba*, *Lemna minor*, *Lemna trisulca*, *Lemna turionifera*, and *Wolffia arrhiza*). The most common duckweed species in the samples from Ukraine were *Le. minor* and *S. polyrhiza*, accounting for 17 and 15 out of 40 specimens, respectively. The most common duckweed species in the samples from China was *S. polyrhiza*, accounting for 15 out of 29 specimens. *La. punctata* and *Le. aequinoctialis* were also common in China, accounting for five and four specimens, respectively. According to both *atpH–atpF* and *psbK–psbI* barcode analyses, the species identified as *Le. aequinoctialis* does not form a uniform taxon similar to other duckweed species, and therefore the phylogenetic status of this species requires further clarification. By monitoring duckweeds using chloroplast DNA sequencing, we not only precisely identified local species and ecotypes, but also provided background for further exploration of native varieties with diverse genetic backgrounds. These data could be useful for future conservation, breeding, and biotechnological applications.

## 1. Introduction

Monitoring and characterizing species biodiversity is essential for germplasm preservation, academic studies, and various practical applications [1]. Duckweed is an important element in aquatic ecosystems worldwide, often covering large portions of the still water surface they inhabit. This group of tiny aquatic plants is composed of 36 species divided into five genera in the Lemnaceae family [2,3], an early diverging family of monocotyledonous plants [4]. 

Duckweeds are a diverse group and provide many opportunities for genetic, physiological, biochemical, and practical research [5,6]. After being important model plants in the 1950s–1970s, duckweeds became popular again in the 2010s, primarily due to their potential as a biofuel feedstock because of their high biomass growth rate, low lignin content, and high starch content [7,8]. In addition to starch, duckweed biomass is rich in proteins, carbohydrates, crude fiber, minerals, and lipids. This biomass composition makes duckweed a potential food source for animals, fish, and humans [9]. Duckweeds have also been studied for their use in wastewater treatment [10,11], biosensing [12,13], and phytoremediation of water reservoirs contaminated with various toxic chemicals [14,15]. Several duckweed species have been genetically engineered with the eventual aim of producing pharmaceutical proteins such as antigens, peptide hormones, and antibodies [16,17,18]. 

The multiple potential applications of duckweed have led to an increasing interest in duckweed genetics, molecular evolution, and diversity. The genome size of duckweeds varies by 14-fold, from 160 Mb in great duckweed (*Spirodela polyrhiza* (L.) Schleid) to ≈2.2 Gb in *Wolffia arrhiza* (L.) Horkel ex Wimm [19]. Researchers have sequenced the whole genomes of two representative ecotypes of *Spirodela polyrhiza* [20,21] and *Spirodela intermedia* W. Koch [22], as well as genomes of *Lemna minor* L. [23] and *Wolffia australiana* (Benth.) Hartog and Plas [24]. Moreover, there are ongoing whole-genome sequencing projects for *Landoltia punctata* (G.Mey.) Les and D.J.Crawford and *Lemna gibba* L. [25]. Four biannual international conferences specifically on duckweed have taken place since 2012, and there has been a tremendous surge in diverse academic and applied studies of various aspects of duckweed biology [5,26,27,28,29]. 

Duckweeds include the smallest known flowering plants and often have reduced morphology, making some species difficult to identify using traditional botanical approaches, not even mentioning ecotypes [30]. Recently, molecular methods have been developed to aid in identifying duckweed species and distinguishing ecotypes [31]. The Consortium for the Barcode of Life (CBOL) [32] recommends seven chloroplast DNA (cpDNA) barcodes to identify land plants simply and reliably [33]. The recommended barcodes have been adapted for identification of duckweeds supported by the constantly growing number of reference sequences deposited in DNA sequence databases. Most of these sequences came from studies of the samples deposited at the world’s largest live duckweed depository at the Rutgers University’s Duckweed Stock Cooperative (RDSC) in New Brunswick, NJ, USA (www.ruduckweed.org, accessed on 31 March 2022), with the rest coming from smaller collections or random samplings. Additionally, 12 chloroplast genome sequences, representing 7 duckweed species [34,35,36,37], have been sequenced and deposited in the NCBI database.

In many parts of the world, including Ukraine and China, farmers practicing intensive agriculture use substantial amounts of fertilizers. Fertilizer that is not fully utilized by crops eventually ends up in water reservoirs surrounding agricultural fields. Due to its ability to quickly assimilate nitrogen, phosphorous, and other nutrients, duckweed can rapidly grow, producing on average of 13–38 dry tons of biomass/hectare/year [38], converting agricultural and municipal wastewater into clean water and a high-value biomass ideal for animal/fish feed and numerous other applications [5]. In both Ukraine and China, duckweed is the dominant vegetation in ponds and lakes. In contrast to China, where different aspects of duckweed research are relatively well developed (for example, the RDSC collection hosts more than 200 duckweed ecotypes originated from China), there is rather scarce information on duckweed in Ukraine and Eastern Europe in general.

In this work, we evaluated the biodiversity of duckweed in different regions of Eastern China and Ukraine on the basis of the two-barcode protocol for sequencing the chloroplast *atpH–atpF* (*ATP*) and *psbK–psbI* (*PSB*) spacers in the collected duckweed specimens. With this approach, we precisely identified local species and ecotypes. Our results provide a foundation for further exploring native varieties with diverse genetic backgrounds and for duckweed breeding and biotechnological applications.

## 2. Results

### 2.1. Genotyping of the Duckweed Specimens

**Figure 1:**
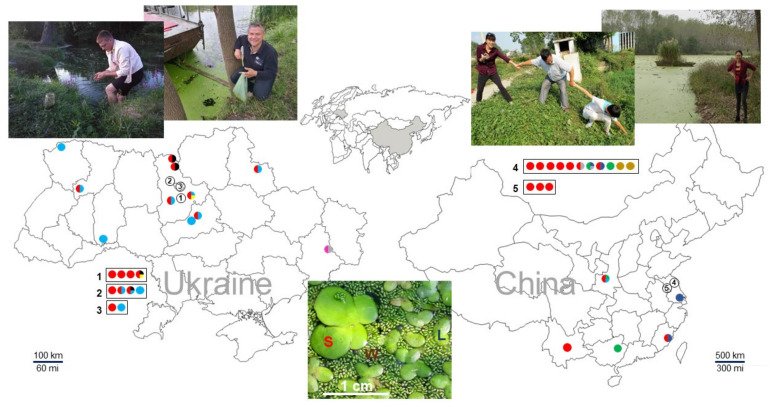
Location of duckweed sampling sites in Ukraine and China.Dot colors correspond to different species: red, *S. polyrhiza*; green, *La. punctata*; light blue, *Le. minor*; dark blue, *Le. aequinoctialis* Welw.; pink, *Le. gibba*; black, *Le. trisulca*; grey, *Le. turionifera*; yellow, *W. arrhiza*; and brown, *W. globosa*. The image at the bottom was taken at a pond in Huai’an, China. It illustrates a community of three different species, *S. polyrhiza* (S), *Le. aequinoctialis* (L), and *W. globosa* (W), growing together. The exact GPS coordinates of the sites are listed in Table S2. Geographic maps were taken from the websites located at https://www.d-maps.com/m/asia/china/chine/chine58.gif (accessed on 11 February 2022) and https://www.d-maps.com/m/europa/ukraine/ukraine50.gif (accessed on 11 February 2022).

We collected 69 duckweed specimens from across Ukraine and southeastern China. Several locations contained more than one species, as illustrated in Figure 1. From these specimens, plus RDSC clone 8656, we obtained 140 representative sequences, which we deposited in GenBank (Table S1). Because the PCR primers for the *atpH–atpF* spacer are located further into the corresponding *atpH* and *atpF* gene sequences compared to *psbK–psbI* spacer, the *ATP* barcodes contain longer portions of the coding sequences compared to *PSB*. The high reliability of the represented barcodes is based on sequences generated using both forward and reverse primers following careful nucleotide validation.

#### 2.1.1. Great Duckweed, *Spirodela polyrhiza*

Barcoding showed that 15 of the 39 specimens collected in Ukraine and 15 of the 30 specimens collected in China were *S. polyrhiza*. The *S. polyrhiza ATP* sequences from our study and the reference sequences from the whole chloroplast genome of U.S. *S. polyrhiza* ecotype 7498 [35,37] had high sequence conservation. The main detected sequence variations were T↔C transitions at defined positions along the sequence and a couple of T↔A transversions, with no biases related to the specimen’s geographic origins (Figure S1A). The *PSB* sequences showed similar low sequence diversity but with different sequence polymorphisms, including single-nucleotide polymorphisms (SNPs), insertion/deletions (InDels), and more random nucleotide transitions/transversions (Figure S1B) compared to the *ATP* sequences. Our *PSB* sequences also contained single-nucleotide insertions of additional A nucleotides at positions 25 and 354 and an additional T at position 402, compared to the reference sequence of *S. polyrhiza* ecotype 7498.

#### 2.1.2. Dotted Duckweed, *Landoltia punctata*

By chloroplast DNA barcoding, we identified five *La. punctata* ecotypes. Two ecotypes were collected near the Hongze lake (Jiangsu province, China) and kept in our *in vitro* collections (NB0014 and NB0022). Ecotype Ya3 was collected from Yanling and Gu1 from Guilin; the ecotype RDSC EL019 collected earlier in Kuhming was obtained from the RDSC (New Brunswick, USA). As *La. punctata* inhabits tropical areas [29], we did not find it in Ukraine. Sequence alignments showed a high stability of both the *ATP* and *PSB* sequences in *La. punctata*. The *ATP* sequences of the six ecotypes only shared two A↔G transitions, both in Gu1, and a single nucleotide deletion (Figure S2A); the six *PSB* sequences differed by a single G→T transversion in Ya3 (Figure S2B).

#### 2.1.3. Common Duckweed, *Lemna minor*

*Lemna minor* was the most represented duckweed species in the Ukraine specimens. We identified 17 of the 39 specimens collected in Ukraine as *Le. minor*. We also identified one specimen from China, Ya2 collected in Yangling, as *Le. minor*. The *ATP* and *PSB* sequences of the *Le. minor* ecotypes had very low sequence divergence, with near 100% similarity to the corresponding 29 and 31 GenBank *ATP* and *PSB* sequences representing *Le. minor* ecotypes, respectively. We compared the sequences of our specimens with the corresponding sequences of a Russian ecotype for which the chloroplast genome was sequenced [34] and found only three nucleotide substitutions in the *PSB* sequences (Figure S3A) and five G→A transition and a single T→G transversion in the *ATP* sequences (Figure S3B).

#### 2.1.4. Star Duckweed, *Lemna trisulca*

We identified four duckweed specimens from Ukraine as *Le. trisulca*. They had a 100% similarity (Figure S4A) to the *ATP* sequences previously reported for ecotypes from the USA and Canada [39]. However, alignment of *PSB* sequences clearly distinguished Ukrainian ecotypes from the North American ones on the basis of the duplication of an AT-rich 23-bp long DNA sequence in the North American ecotypes (Figure S4B). Moreover, alignment of a few *Le. trisulca ATP* and *PSB* sequences [39] revealed distinct variants in strain UTCC 399 of unknown origin, characterized by short 4–6-nucleotide insertions/deletions as compared to the Ukrainian and North American ecotypes.

#### 2.1.5. Turion Duckweed, *Lemna turionifera*

We identified one specimen from Ukraine (from the southeast) and two collected from China (from near Hongze lake) as *Le. turionifera*. The *ATP* and *PSB* sequences of these specimens showed no sequence variation when aligned with *Le. turionifera* sequences from Canada, the Czech Republic, and Lake Tai in China [40]. The only variation we found was a single nucleotide deletion in the *ATP* sequence of the accession from the Czech Republic (Figure S5).

#### 2.1.6. Swollen or Fat Duckweed, *Lemna gibba*

We identified one specimen from Ukraine (DW102) as *Le. gibba*. The *PSB* sequence showed homology with the corresponding sequences of four *Le. gibba* strains from the USA, Italy, Ethiopia, and Japan [39], as well as strain RDSC 5504, which originated from Shanghai, China. The *ATP* sequence of DW102 differed in two positions, a single insertion of A (which was also in the sequence of the Shanghai strain) and a unique C→T transition (Figure S6).

#### 2.1.7. Lesser Duckweed, *Lemna aequinoctialis*

*Lemna aequinoctialis* had the highest variation in *ATP* and *PSB* sequences among the species analyzed in this study. We collected four *Le. aequinoctialis* strains: two from Huai’an city (I2 and NB0017), one from Shanghai (NB0007), and one from Fuzhou (Fu94). We divided the strains into two groups on the basis of their barcode sequences (Figure S7). Strains NB0017 and NB0007 differed from I2 and Fu94 by two tandem duplications of 21 and 5 bp, three specific SNPs in their *PSB* sequences (positions 71, 117, and 162), and five SNPs (positions 12, 189, 359, 364, and 395) in their *ATP* sequences. These two groups aligned with three American *Le. aequinoctialis* strains [39], with NB0017 and NB0007 having similar sequences to those of strains 6612 (Centerville, CA, USA) and 8656 (Argentina), whereas I2 and Fu94 aligned with strains 6746 (Plainsburg, CA, USA) and 7126 (Texas, USA) (Figure S7). All four Chinese strains had three specific SNPs in their *PSB* sequences (positions 357, 401, 444) compared to the three American strains.

**Figure 2:**
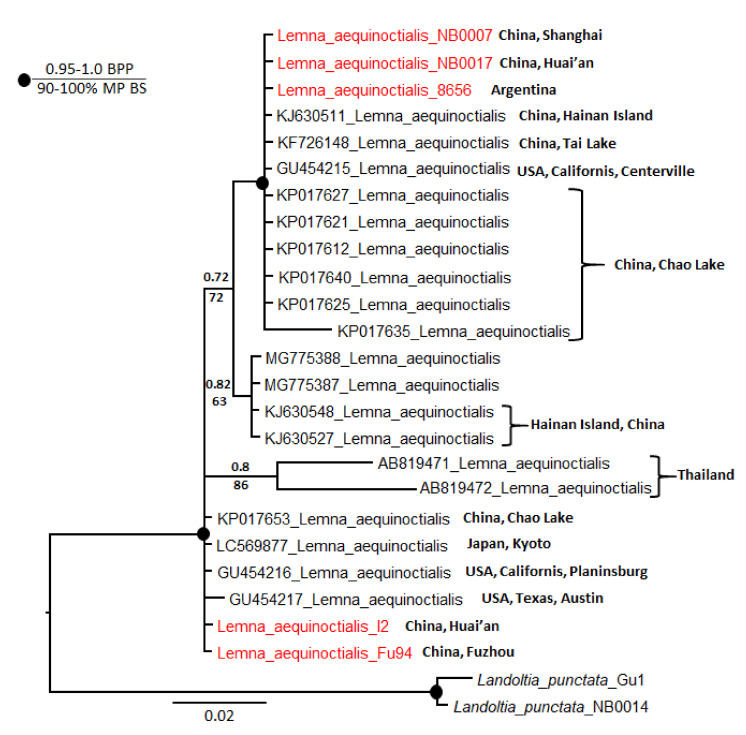
Bayesian consensus tree based on analysis of *atpH–atpF* intergenic spacer sequences of *Lemna aequinoctialis* and *Landoltia punctata* as an outgroup. Bayesian posterior probabilities (BPP) and maximum parsimony bootstrap values (MP BS) are shown above and below the branches, respectively. Strongly supported clades (MP BS > 90% and BPP > 0.95) are indicated with black circles at the branchpoints. For the origin of specimens, see Table S2.

To examine polymorphism in *Le. aequinoctialis* barcodes in more detail, we analyzed the phylogeny of the 21 *ATP* sequences available in NCBI GenBank together with our five sequences. We included two *La. punctata* accessions as an outgroup. In total, there were 513 characters, of which 453 were constant. Of the variable characters, 18 were parsimony uninformative and 42 were parsimony informative. Parsimony and Bayesian analyses yielded the same topology but with lower bootstrap percentages than posterior probabilities. A heuristic search found most-parsimonious trees that were 70 steps long (consistency index 0.9143, retention index 0.9259). The resultant dendrogram from this analysis is shown in Figure 2. All sequences were divided into four subclades: two with very strong support and two with little support. Our accessions are subordinate to the two strongly supported clades.

#### 2.1.8. Least Duckweed, *Wolffia arrhiza*, and Watermeal Duckweed, *Wolffia globosa*

We identified two Ukrainian specimens as *W. arrhiza* and two Chinese specimens as *W. globosa*. Both specimens from Ukraine, DW32 and DW35, had high *ATP* sequence similarity with the homologous sequence of African and Italian specimens, but a high level of nucleotide mismatches with the sequence from a *W. arrhiza* specimens from Brazil (Figure S8A). There was 100% similarity between the *PSB* sequences of the two Ukrainian specimens, with the sequence blasting revealing a single hit in GenBank (Figure S8B).

There were more hits for *W. globosa* compared to *W. arrhiza*; 31 for *ATP* and 14 for *PSB*. The Chinese strains C2 and NB0015 (characterized in this study), together with strains DW2101-4 (Acc. KJ630544.1; Hainan) and LC49 (Lake Chao) [41], were more closely grouped with a specimen from the USA [39] than with those from other Asian countries India, Japan, and Thailand (Figure S9). This grouping was based on nucleotide substitutions at positions 248, 253, and 383 in the *ATP* sequence (Figure S9A) and, even more profoundly, by multiple SNPs and three deletions/insertions of short nucleotide sequences in the *PSB* sequence (Figure S9B).

### 2.2. Phylogenetic Analysis

Phylogenetic analysis of our 69 duckweed specimens using *ATP* and *PSB* sequences separately showed no conclusive results. Therefore, we performed a combined *ATP* and *PSB* analysis of 70 taxa, including *Pistia stratiotes* [42] as an outgroup species. The combined data matrix included 1197 characters divided in two partitions: 1–560 for *ATP* and 561–1197 for *PSB*, of which 751 were constant, 103 were parsimony uninformative, and 343 were parsimony informative.

**Figure 3:**
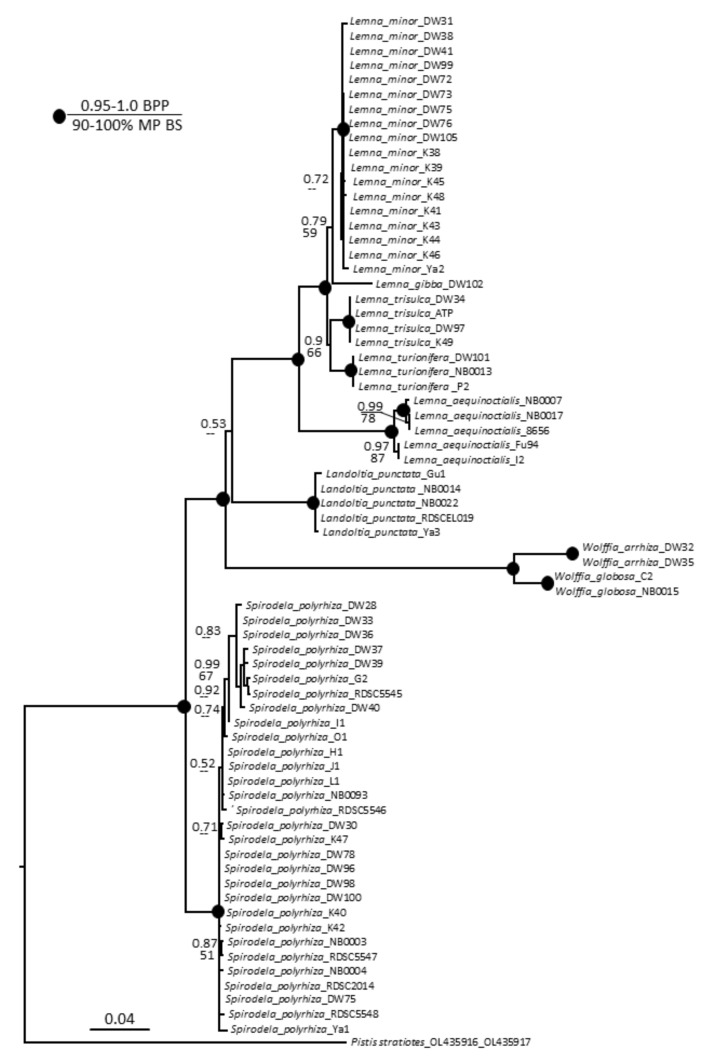
Bayesian consensus tree based on analysis of the combined chloroplast DNA dataset (*atpH–atpF* and *psbK–psbI* intergenic spacers) of Lemnaceae taxa and *Pistia* *stratiotes* as an outgroup. Bayesian posterior probabilities (BPP, top value) and maximum parsimony bootstrap values (MP BS, bottom value) are shown at the branches. Strongly supported clades (MP BS > 90% and BPP > 0.95) are indicated with black circles at the branchpoints.

Parsimony and Bayesian analyses yielded the same topology but with lower bootstrap percentages than posterior probabilities. The heuristic search found most-parsimonious trees that were 635 steps long (consistency index 0.8551, retention index 0.9691). The resultant dendrogram from this analysis is shown in Figure 3. All species studied built monophyletic and mostly not polymorphic clades, with few exceptions. The *S. polyrhiza* clade had several small subclades with weak support, and the clade with *L. aequinoctialis* accessions was divided into two subclades with strong support. Overall, this phylogeny of Lemnaceae is congruent with previous studies [3,43,44,45].

## 3. Discussion

**Figure 4:**
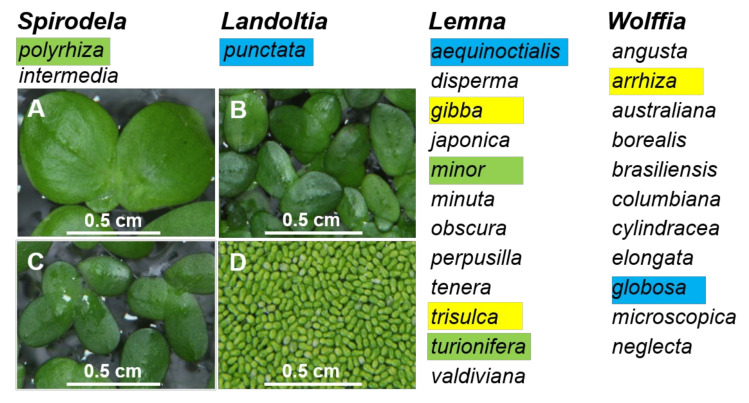
Four genera of the Lemnaceae plant family were represented by duckweed species in Ukraine and China. Species that were found in Ukraine and China are highlighted green, those found only in Ukraine are highlighted yellow, and those found only in China are highlighted blue. A, B, C, and D are representative images of *Spirodela polyrhiza, Landoltia punctata, Lemna minor*, and *Wolffia globose*, respectively, from the in vitro collection of Huaiyin Normal University.

As the smallest known flowering plants, duckweeds have a reduced morphology, which makes them difficult to identify using traditional botanical approaches [30]. Therefore, molecular approaches [2] offer valuable alternatives for species monitoring of this ancient group of plants. Here, to provide additional data supporting molecular approaches for duckweed identification, as well as to examine duckweed diversity, we collected 39 duckweed specimens from Ukraine and 30 from China. Using DNA barcoding, we identified six duckweed species among the Ukrainian specimens (*S. polyrhiza, Le. gibba, Le. minor, Le. trisulca, Le. turionifera*, and *W. arrhiza*) and six species from China (*S. polyrhiza, La. punctata, Le. aequinoctialis, Le. minor, Le. turionifera*, and *W. globosa*). These species represent four out of the five genera in the Lemnaceae family (Figure 4). The only genus not represented was *Wolffiella*, which only occurs in the Americas and Africa [29].

The distribution of duckweed species in this study generally matched previously identified duckweed distributions, with *S. polyrhiza*, *Le. Minor*, and *W. arrhiza* being the most common species in Europe and *S. polyrhiza*, *La. Punctate*, *Le. aequinoctialis*, and *W. globoza* the usual species in China [29,46]. However, there is little information on duckweed biodiversity in Eastern Europe in general and in Ukraine in particular [47,48]. To the best of our knowledge, this study is the first chloroplast-barcoding-based record of duckweed biodiversity in an Eastern European country. Compared to Ukraine, duckweed biodiversity in China is relatively well investigated [31,40,41], and there are numerous ecotypes from China deposited in the RDSC world collection in the USA (New Brunswick, NJ) and in different institutions in China [49,50].

Our molecular identification of randomly sampled specimens agrees that the great duckweed, *S. polyrhiza*, is the most cosmopolitan of the 36 duckweed species recognized worldwide [29]. It was the most dominant species in East China and in Ukraine. Although the phylogenetic analysis demonstrated a certain degree of clustering of the *S*. *polyrhiza* specimens on the basis of the limited barcode sequence variations (Figure 3), it did not show any clear links to the geographic origin of the specimens. Generally, the *ATP* and *PSB* sequences of our specimens had almost 100% similarity with the corresponding sequences of strain 7498 [37], with a low sequence variability between the specimens. Similarly, *S. polyrhiza* nuclear genomes sequenced from 63 specimens collected worldwide had high sequence conservation [51].

*La. punctata* is the only representative of the genus *Landoltia*. It is considered to be closely related to *Spirodela* [36] but is not as widely distributed as *S. polyrhiza*. It mostly inhabits tropical and subtropical areas [29]. Therefore, it is not surprising that we collected *La. punctata* specimens in China but none in Ukraine. Genetic analysis of the six Chinese *La. punctata* specimens revealed few nucleotide substitutions. This stability of ATP and PSB sequences was also observed among specimens from different geographic origins, including India, Africa, America, and Australia (Figure S2).

We identified five *Lemna* species among the specimens collected from Ukraine and China. The common duckweed, *Le. minor*, was the most predominant duckweed species in Ukraine, represented by 17 out of 40 specimens, closely followed by *S. polyrhiza* (15 specimens) (Figure 1). However, we only identified one specimen from China as *Le. minor*, a specimen that was collected in north-central China. *Lemna turionifera* was a minor species in both China and Ukraine [40,45]. We identified *Le. gibba* and *Le. trisulca* only in Ukraine, and *Le. aequinoctialis* only in China (Figure 1).

Three *Lemna* species, *Le. minor, Le. gibba*, and *Le. turionifera*, have stable species-specific *ATP* and *PSB* sequences as reflected in the phylogenetic dendrogram (Figure 3), with very few variations compared to their counterparts from other parts of the world available in GenBank. The three *Le. trisulca* specimens from Ukraine had perfect *ATP* sequence similarity; however, there were clear differences in *PSB* sequences compared (Figure S4) with specimens from the USA and Canada [39]. The differences were due to a 23 bp duplication.

The most intriguing results from this study were on the phylogeny of *Le. aequinoctialis*. On the basis of the *ATP* and *PSB* sequences, we grouped the four *Le. aequinoctialis* specimens (collected in Fuzhou, Shanghai, and Huai’an) into two clades (Figure S7). Sequences of these clades were aligned with the *ATP* sequences of *Le. aequinoctialis* strain DW0101-3 (Hainan, China; Acc. KJ630511.1) and strain LC42 (Lake Chao, China) [41]. We constructed a phylogeny based on all *Le. aequinoctialis ATP* sequences in GenBank, and the resulting tree was complex (Figure 2). On the basis of these results, we suggest that the status of this species needs further careful examination.

We identified two *Wolffia* species in our study: *W. arrhiza*, a common species in Europe and Africa, and *W. globosa*, which inhabits Southeast Asia [29], were identified in Ukraine and China, respectively. The *W. arrhiza* sequences showed high sequence similarity with other *W. arrhiza* sequences of specimens from Europe and Africa (Figure S8). The *W. globosa* sequences had several characteristic SNPs, both in the ATP and PSB sequences, compared with sequences of specimens from India, Japan, and Thailand (Figure S9); however, they had a higher similarity to sequences of specimens from the USA [39]. The USA specimen is likely a recent invasion in addition to the native *Wolffia columbiana* [29].

## 4. Materials and Methods

### 4.1. Plant Material

Duckweed specimens were collected from various still water reservoirs, lakes, and ponds across eastern China and Ukraine during 2016–2019. Prior to genotyping, most specimens from China were sterilized and kept under aseptic conditions on agar medium according to previously described methods [43]. These specimens were kept at the duckweed *in vitro* collection recently organized at Huaiyin Normal University, Hui’an, China [47], barcoded and used in our previous studies [11,18,52,53]. The Ukrainian duckweed specimens were collected from water reservoirs, sorted according to their morphological characteristics, and directly subjected to DNA extraction for further chloroplast DNA barcoding. All analyzed duckweed specimens and locations are listed in Table S2.

### 4.2. DNA Extraction, Fragment Amplification, Sequencing, and Alignment

Total DNA was extracted from plant tissue using a modified CTAB method [54]. The PCR amplifications were carried out as recommended by the CBOL Plant Working Group [32], described in [39], using primers 5′-TTAGCATTTGTTTGGCAAG and 5′-AAAGTTTGAGAGTAAGCAT for the *psbK–psbI* intergenic spacer and 5′-ACTCGCACACACTCCCTTTCC and 5′-GCTTTTATGGAAGCTTTAACAAT for the *atpH–atpF* intergenic spacer. Following amplification, the DNA fragments were sent to Sangon Biotech (Shanghai, China) for purification and sequencing. The raw sequences were preliminarily optimized using the ‘Online Analysis Tools’ package (http://molbiol-tools.ca, accessed on 25 March 2022), in particular, the program MAFFT, version 7 (https://mafft.cbrc.jp/alignment/server/ accessed on 25 March 2022). Multiple DNA sequence alignments were generated with ClustalW software [55], and the alignments were subsequently corrected manually in MEGA 5 [56].

For BLAST alignment analyses, a duckweed reference barcode set was compiled from *ATP* and *PSB* sequences that were generated in this study and those available from the NCBI database as of January 2022. Queried sequences were trimmed to include only intergenic regions and used in BLASTN (version 2.2.26+) searches to identify homologies to other barcode sequences in the set. The number of top hits for each query are presented in Table S1.

### 4.3. Phylogenetic Analysis

Phylogenetic analysis was carried out on individual and combined *ATP* and *PSB* sequences using parsimony and Bayesian methods. *Pistia stratiotes*, from the Araceae family, was used as an outgroup for the Lemnaceae family. Parsimony analysis was performed with PAUP* 4.0b10 [57] using heuristic searches with tree bisection–reconnection and 100 random additional sequence replicates. Bootstrap support (BS) [58] was estimated with 100 bootstrap replicates, each with 100 random addition sequence searches. Bayesian analyses were implemented with MrBayes 3.1.23 [59]. Sequence evolution models were evaluated using the Akaike information criterion (AIC) with the aid of jModelTest2 v2.1.6 [60]. Two independent runs each of eight chains and 10 million generations, sampling every 1000 generations, were executed, and 25% of initial trees were discarded as burn-in. The remaining 15,000 trees were combined into a 50% majority-rule consensus tree.

## 5. Conclusions

Our survey of the duckweed species in Ukraine and China makes a solid contribution to monitoring the biodiversity of aquatic flora in these countries. In addition to precisely identifying six major species and their geographic distribution in each these countries by double chloroplast DNA barcoding, our data highlighted the need to re-examine the phylogenetic status of one of those species, *Lemna aequinoctialis*. The study added 138 new chloroplast *ATP* and *PSB* barcodes to the 1754 corresponding barcodes for these species available in the NCBI database as of March 2022 (Table S1). These new resources might fuel future research on plant molecular evolution, biodiversity conservation, breeding, and various biotechnological applications.

## Data Availability

GenBank accession numbers for the *atpF–atpH* (*ATP*) and *psbK–psbL* (*PSB*) barcodes are in the Table S1.

## Supplementary Materials

The following supporting information can be downloaded at: https://www.mdpi.com/article/10.3390/plants11111468/s1, Figure S1: Nucleotide alignment of *atpH–atpF* and *psbK–psbI* spacers sequences of *Spirodela polyrhiza* collected in Ukraine and China (for ecotype details see supplemental Table S2). Matching residues are shown as dots. A. Nucleotide alignment of *atpH–atpF* barcodes including representative sequences of *S. polyrhiza* specimens available in GenBank: MN419335, USA; GU454201, Hong Kong; GU454202, India; GU454203, Malaysia; GU454204, USA; GU454205, Mexico; GU454206, Canada; GU454208, Europe. B. Nucleotide alignment of *psbK-psbI* barcodes including representative sequences of *S. polyrhiza* specimens available in GenBank: MN419335, USA; GU454297, Hong Kong; GU454298, India; GU454299, Malaysia; GU454300, USA; GU454301, Mexico; GU454302, Canada; GU454304, Europe. Figure S2: Nucleotide alignment of *atpH-atpF* and *psbK-psbI* spacers of *Landoltia punctata* (for ecotypes details see supplemental Table S2). Matching residues are shown as dots. A. Nucleotide alignment of *atpH-atpF* barcodes including the representative sequences of *La. punctata* from different parts of the world available in the GenBank: GU454209, South Africa; GU454211, India; GU454212, USA; GU454213, Australia. B. Nucleotide alignment of *psbK-psbI* barcodes including the representative sequences of *La. punctata* from different parts of the world available in the GenBank: GU454305, South Africa; GU454307, India; GU454308, USA; GU454309, Australia. Figure S3: Nucleotide alignment of *atpH-atpF* and *psbK-psbI* spacers of *Lemna minor* (for ecotypes details see supplemental Table S2). Matching residues are shown as dots. A. Nucleotide alignment of *atpH-atpF* barcodes including the representative sequences of *Le. minor* from different parts of the world available in the GenBank: DQ400350, Russia; GU454226, Turkey; GU454227, USA; GU454228, South Africa; GU454229, Japan; GU454230, Finland; GU454231, Germany. B. Nucleotide alignment of *psbK-psbI* barcodes including the representative sequences of *Le. minor* from different parts of the world available in the GenBank: DQ400350, Russia; GU454322, Turkey; GU454323, USA; GU454324, South Africa; GU454325, Japan; GU454326, Finland; GU454327, Germany. Figure S4: Nucleotide alignment of *atpH-atpF* and *psbK-psbI* spacers of *Lemna trisulca* (for ecotypes details see supplemental Table S2). Matching residues are shown as dots. A. Nucleotide alignment of *atpH-atpF* barcodes including the representative sequences of *Le. trisulca* from different parts of the world available in the GenBank: GU454236, Canada; GU454237, USA; GU454237, UTCC 399. B. Nucleotide alignment of *psbK-psbI* barcodes including the representative sequences of L. trisulca from different parts of the world available in the GenBank: GU454332, Canada; GU454333, USA; GU454334, UTCC 399. Figure S5: Nucleotide alignment of *atpH-atpF* and *psbK-psbI* spacers of *Lemna turionifera* (for ecotypes details see supplemental Table S2). Matching residues are shown as dots. A. Nucleotide alignment of *atpH-atpF* barcodes including the representative sequences of *Le. turionifera* from different parts of the world available in the GenBank: KF726146, China; MG000422, Canada; GU454240, Czech. B. Nucleotide alignment of *psbK-psbI* barcodes including the representative sequences of *Le. turionifera* from different parts of the world available in the GenBank: MG000496, Canada; GU454335, China; GU454336, Czech. Figure S6: Nucleotide alignment of *atpH-atpF* and *psbK-psbI* spacers of *Lemna gibba*, ecotype DW102 (Supplemental Table S2). Matching residues are shown as dots. A. Nucleotide alignment of the DW102 *atpH-atpF* barcode with the representative sequences of *Le. gibba* from different parts of the world available in the GenBank: KX212889, China; GU454219, USA; GU454220, Italy; GU454221, Ethiopia; GU454222, Japan. B. Nucleotide alignment of the DW102 *psbK-psbI* barcode with the representative sequences of *Le. gibba* from different parts of the world available in the GenBank: GU454315, USA; GU454316, Italy; GU454317, Ethiopia; GU454318, Japan. Figure S7: Nucleotide alignment of *atpH-atpF* and *psbK-psbI* spacers of *Lemna aequinoctialis* (for ecotypes details see supplemental Table S2). Matching residues are shown as dots. A. Nucleotide alignment of *atpH-atpF* barcodes including the representative sequences of *Le. aequinoctialis* from different parts of the world available in the GenBank: GU454215, USA, California, Centerville; GU454216, USA, California, Plainsburg; GU454217, USA, Texas, Austin. B. Nucleotide alignment of *psbK-psbI* barcodes including the representative sequences of *Le. aequinoctialis* from different parts of the world available in the GenBank: GU454311, USA, California, Centerville; GU454312, USA, California, Plainsburg; GU454313, USA, Texas, Austin. Figure S8: Nucleotide alignment of *atpH-atpF* and *psbK-psbI* spacers of *Wolffia arrhiza* (for ecotypes details see supplemental Table S2). Matching residues are shown as dots. A. Nucleotide alignment of *atpH-atpF* barcodes including the representative sequences of *W. arrhiza* from different parts of the world available in the GenBank: MG812317, Kenya; MG775412, Italy; MG775413, Brazil. B. Nucleotide alignment of *psbK-psbI* barcodes including the representative sequence of *W. arrhiza* available in the GenBank: GU454364, Hungary. Figure S9: Nucleotide alignment of *atpH-atpF* and *psbK-psbI* spacers of *Wolffia globosa* (for ecotypes details see supplemental Table S2). Matching residues are shown as dots. A. Nucleotide alignment of *atpH-atpF* barcodes including the representative sequences of *W. globosa* from different parts of the world available in the GenBank: GU454281, USA; KJ630544, China; KP017660, China; MG812325, India; MN881100, Japan. B. Nucleotide alignment of *psbK-psbI* barcodes including the representative sequences of *W. globosa* from different parts of the world available in the GenBank: GU454378, USA; GU454379, Thailand; MG812330, India; MN881100, Japan. References [61,62,63,64,65,66,67,68,69,70,71] are cited in the supplementary materials.
