# Supplementary material for: Biodiversity of Duckweed (Lemnaceae) in Water Reservoirs of Ukraine and China Assessed by Chloroplast DNA Barcoding
Source: Plants (Basel). 2022 May 30;11(11):1468. doi: 10.3390/plants11111468 (PMC9182681; doi:10.3390/plants11111468)
Supplement: Supplementary file 1 [file plants-11-01468-s001.zip › plants-1691397-SI/Proofred- Chen et al.-Supplemental Figures.pdf]

S1A

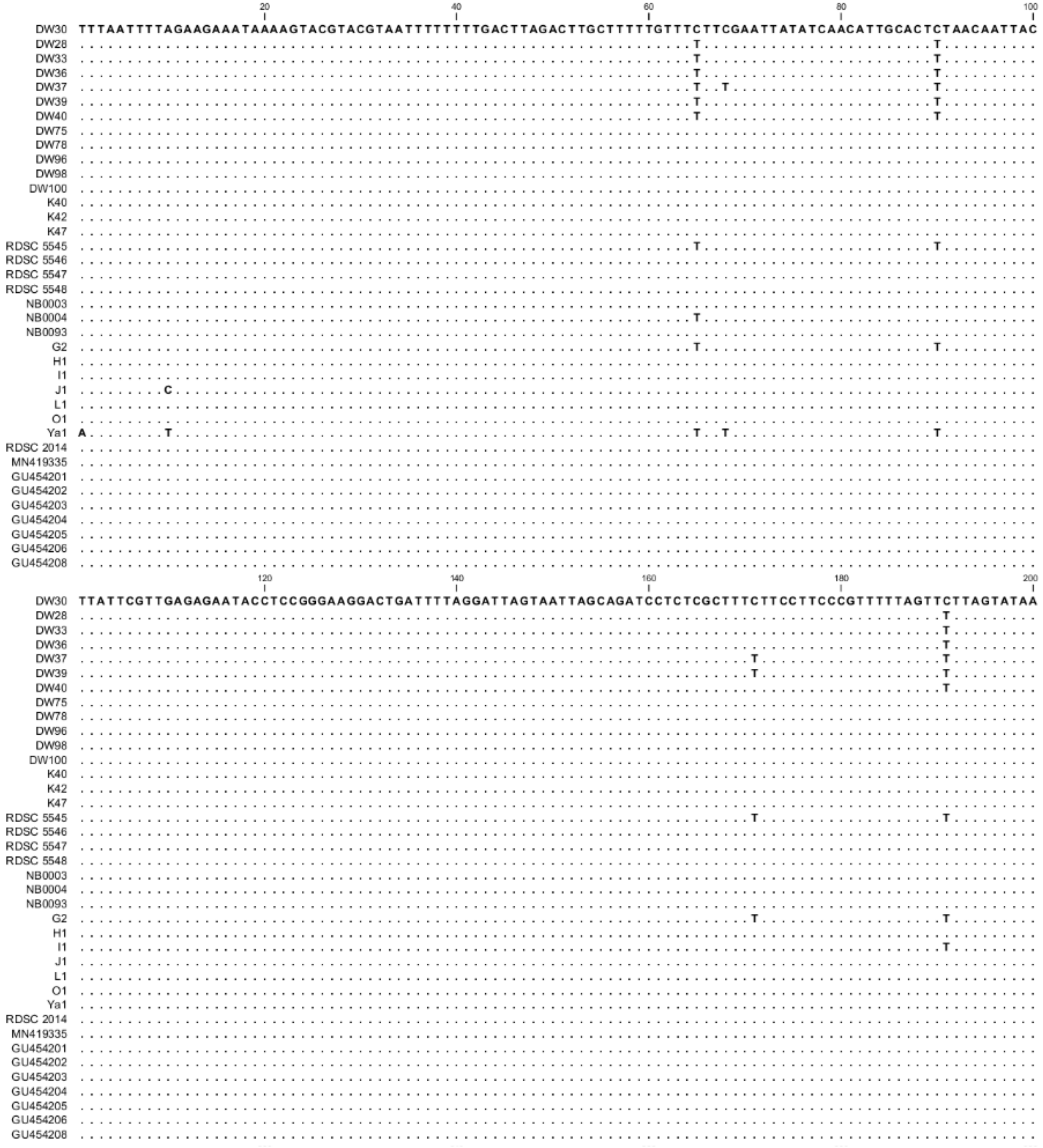

20 40 60 80 100
DW30 TTTAATTTTAGAAGAAATAAAAGTACGTACGTAATTTTTTTTGACTTAGACTTGCTTTTTGTTTCTTCGAATTATATCAACATTGCACTCTAACAATTAC
120 140 160 180 200
DW30 TTATTCGTTGAGAGAATACCTCCGGGAAGGACTGATTTTAGGATTAGTAATTAGCAGATCCTCTCGCTTTCTTCCTTCCCGTTTTTAGTTCTTAGTATAA
DW28
DW33
DW36
DW37
DW39
DW40
DW75
DW78
DW96
DW98
DW100
K40
K42
K47
RDSC 5545
RDSC 5546
RDSC 5547
RDSC 5548
NB0003
NB0004
NB0093
G2
H1
I1
J1
L1
O1
Ya1
RDSC 2014
MN419335
GU454201
GU454202
GU454203
GU454204
GU454205
GU454206
GU454208

S1A

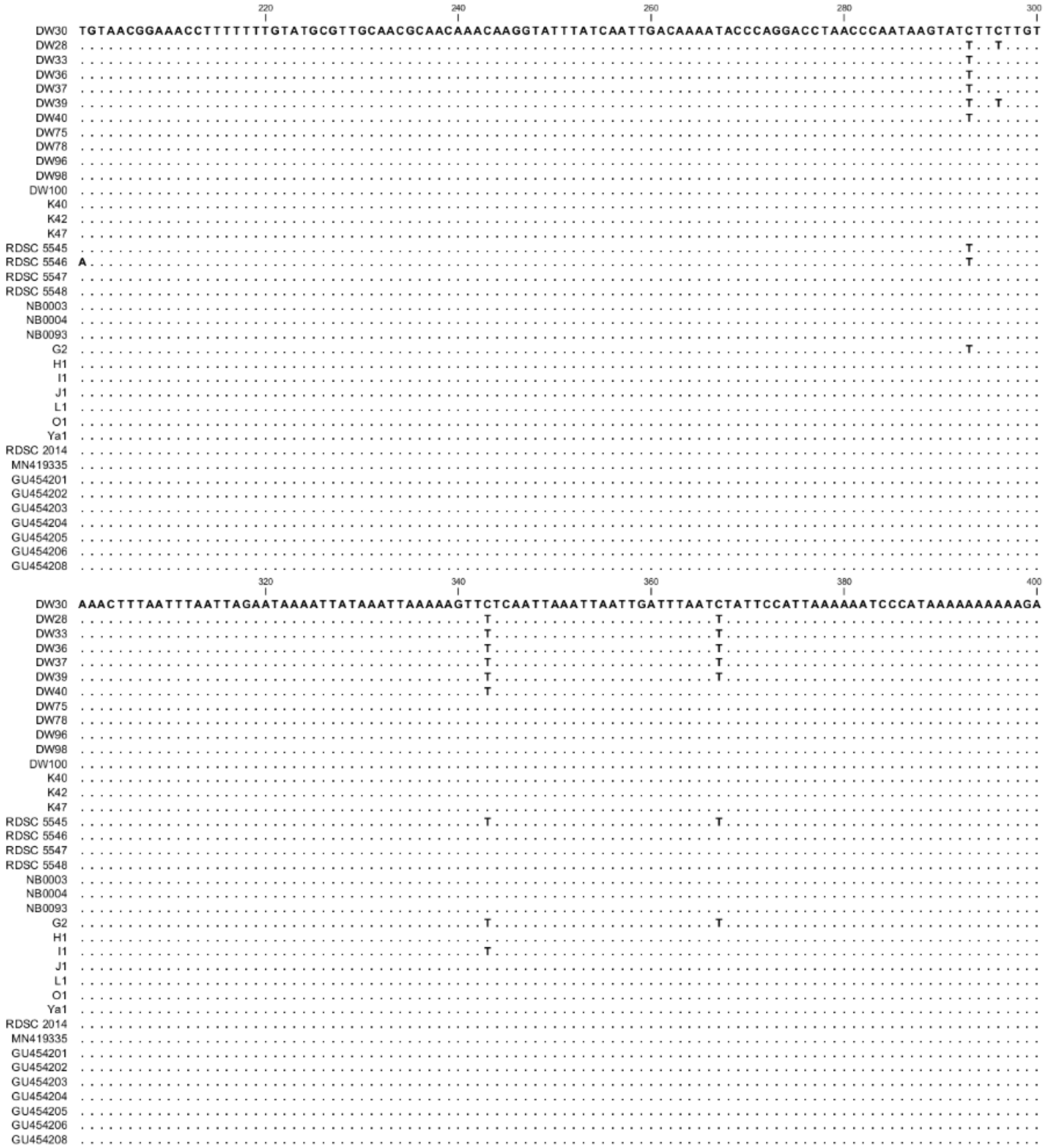
220
240
260
280
300
320
340
360
380
400
DW30
DW28
DW33
DW36
DW37
DW39
DW40
DW75
DW78
DW96
DW98
DW100
K40
K42
K47
RDSC 5545
RDSC 5546
RDSC 5547
RDSC 5548
NB0003
NB0004
NB0093
G2
H1
I1
J1
L1
O1
Ya1
RDSC 2014
MN419335
GU454201
GU454202
GU454203
GU454204
GU454205
GU454206
GU454208

S1A

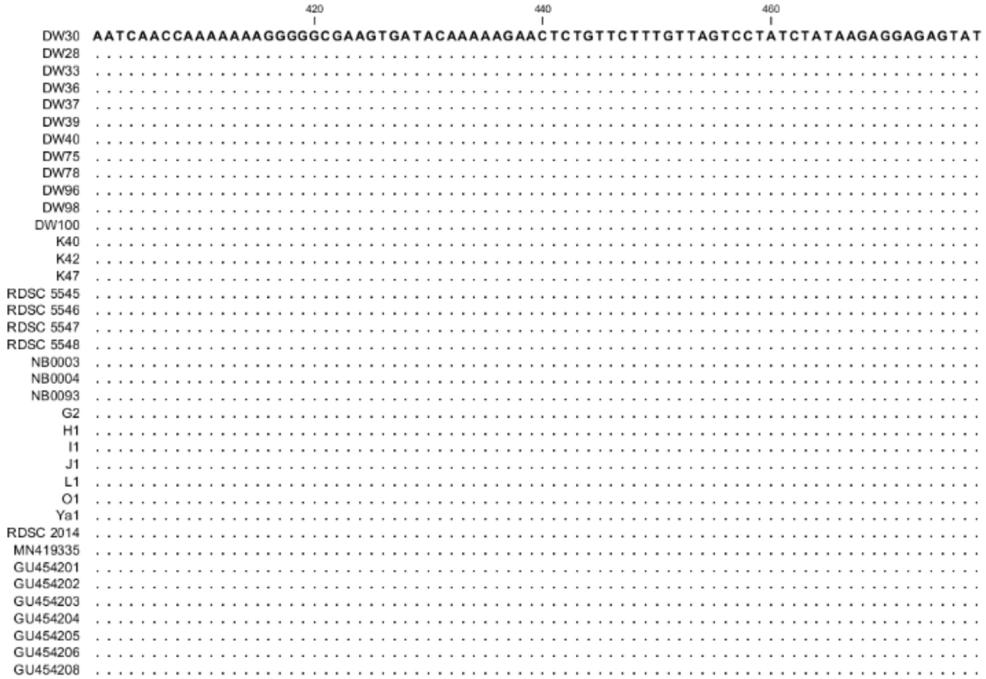
420
440
460
DW30 AATCAACCAAAAAAAAGGGGGCGAAGTGATACAAAAAGAACTCTGTTCTTTGTTAGTCCTATCTATAAGAGGAGAGTAT
DW28
DW33
DW36
DW37
DW39
DW40
DW75
DW78
DW96
DW98
DW100
K40
K42
K47
RDSC 5545
RDSC 5546
RDSC 5547
RDSC 5548
NB0003
NB0004
NB0093
G2
H1
I1
J1
L1
O1
Ya1
RDSC 2014
MN419335
GU454201
GU454202
GU454203
GU454204
GU454205
GU454206
GU454208

S1B

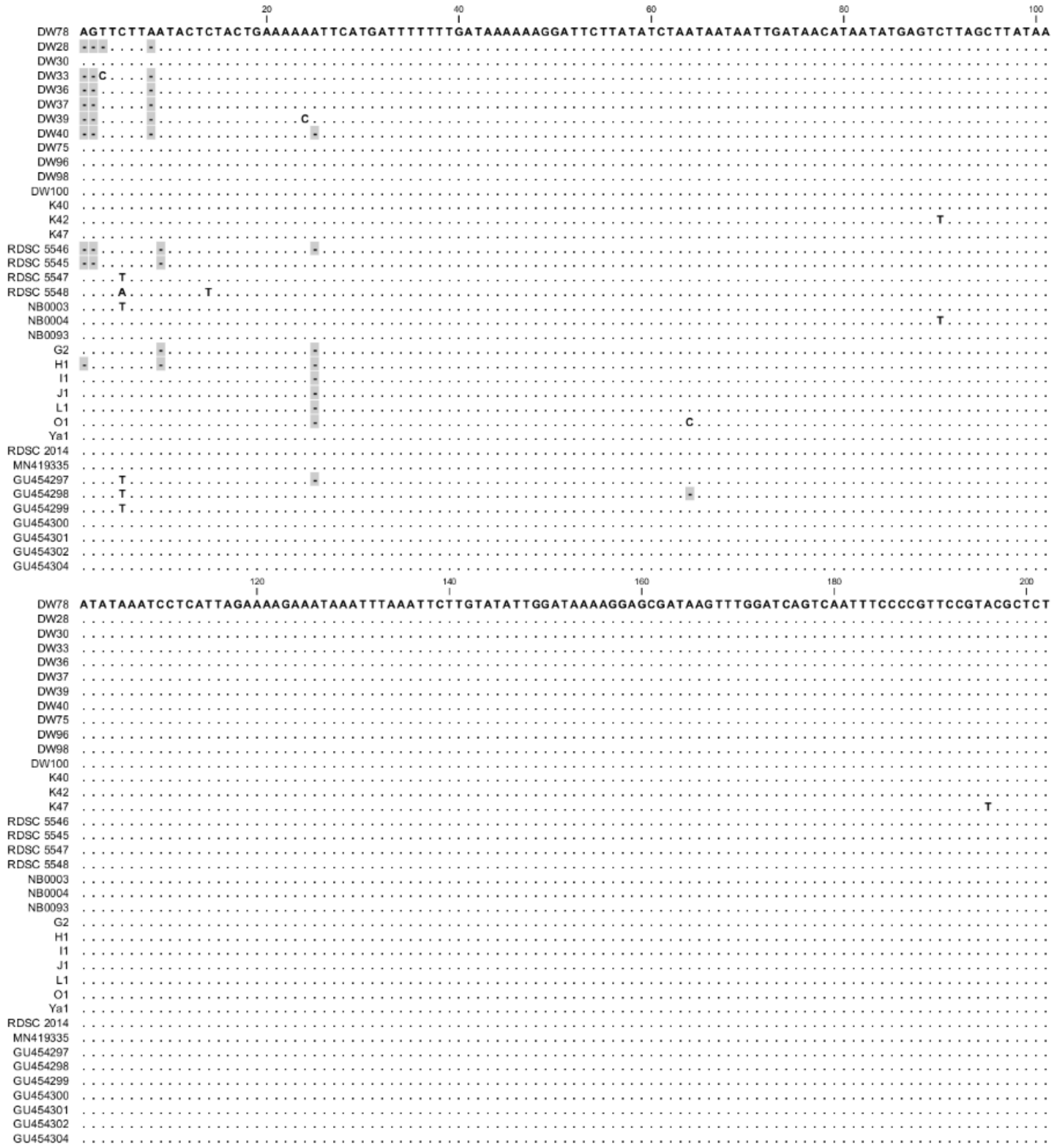
20
40
60
80
100
DW78
DW28
DW30
DW33
DW36
DW37
DW39
DW40
DW75
DW96
DW98
DW100
K40
K42
K47
RDSC 5546
RDSC 5545
RDSC 5547
RDSC 5548
NB0003
NB0004
NB0093
G2
H1
I1
J1
L1
O1
Ya1
RDSC 2014
MN419335
GU454297
GU454298
GU454299
GU454300
GU454301
GU454302
GU454304
120
140
160
180
200

S1B

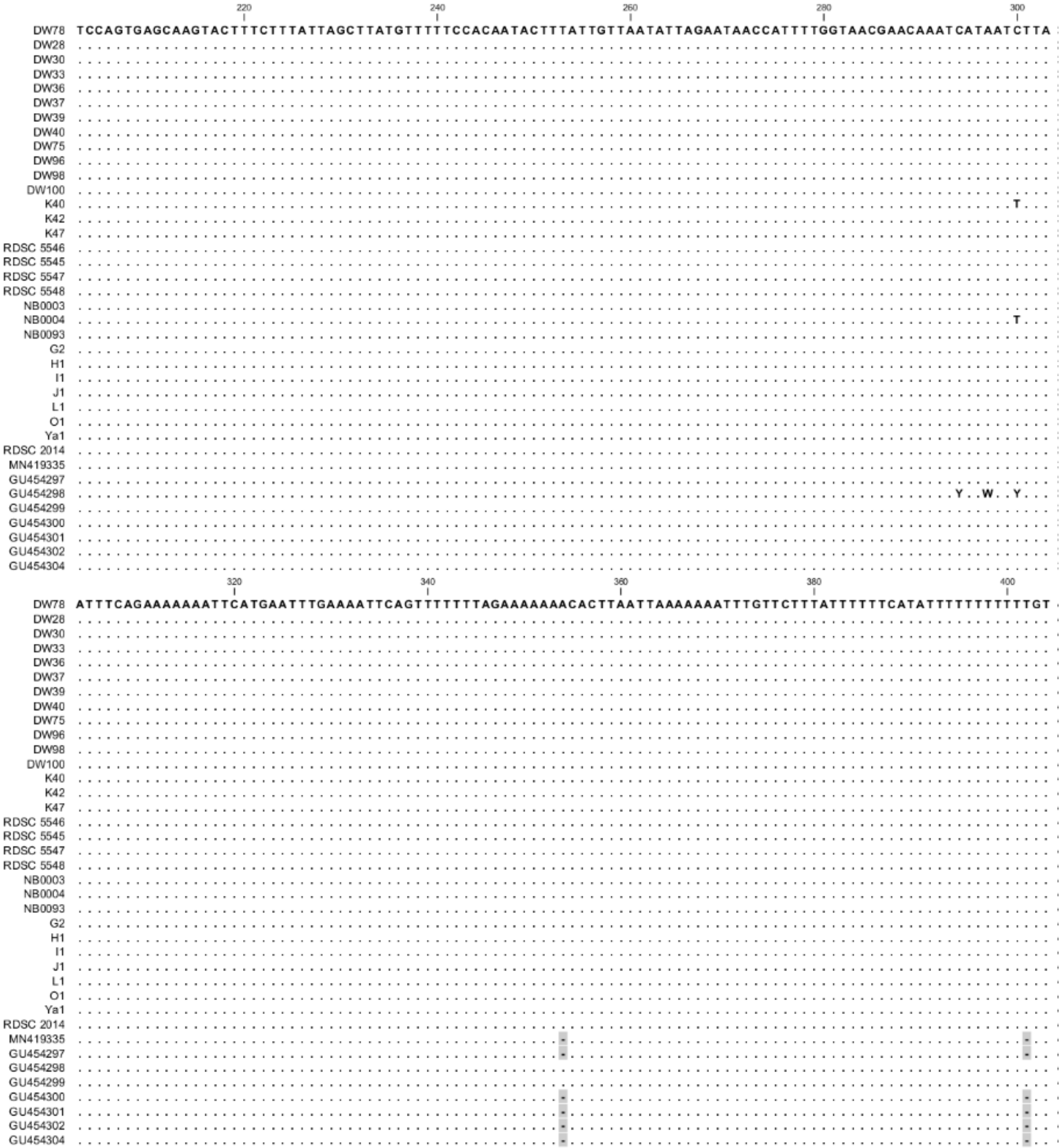
DW78
DW28
DW30
DW33
DW36
DW37
DW39
DW40
DW75
DW96
DW98
DW100
K40
K42
K47
RDSC 5546
RDSC 5545
RDSC 5547
RDSC 5548
NB0003
NB0004
NB0093
G2
H1
I1
J1
L1
O1
Ya1
RDSC 2014
MN419335
GU454297
GU454298
GU454299
GU454300
GU454301
GU454302
GU454304

S1B

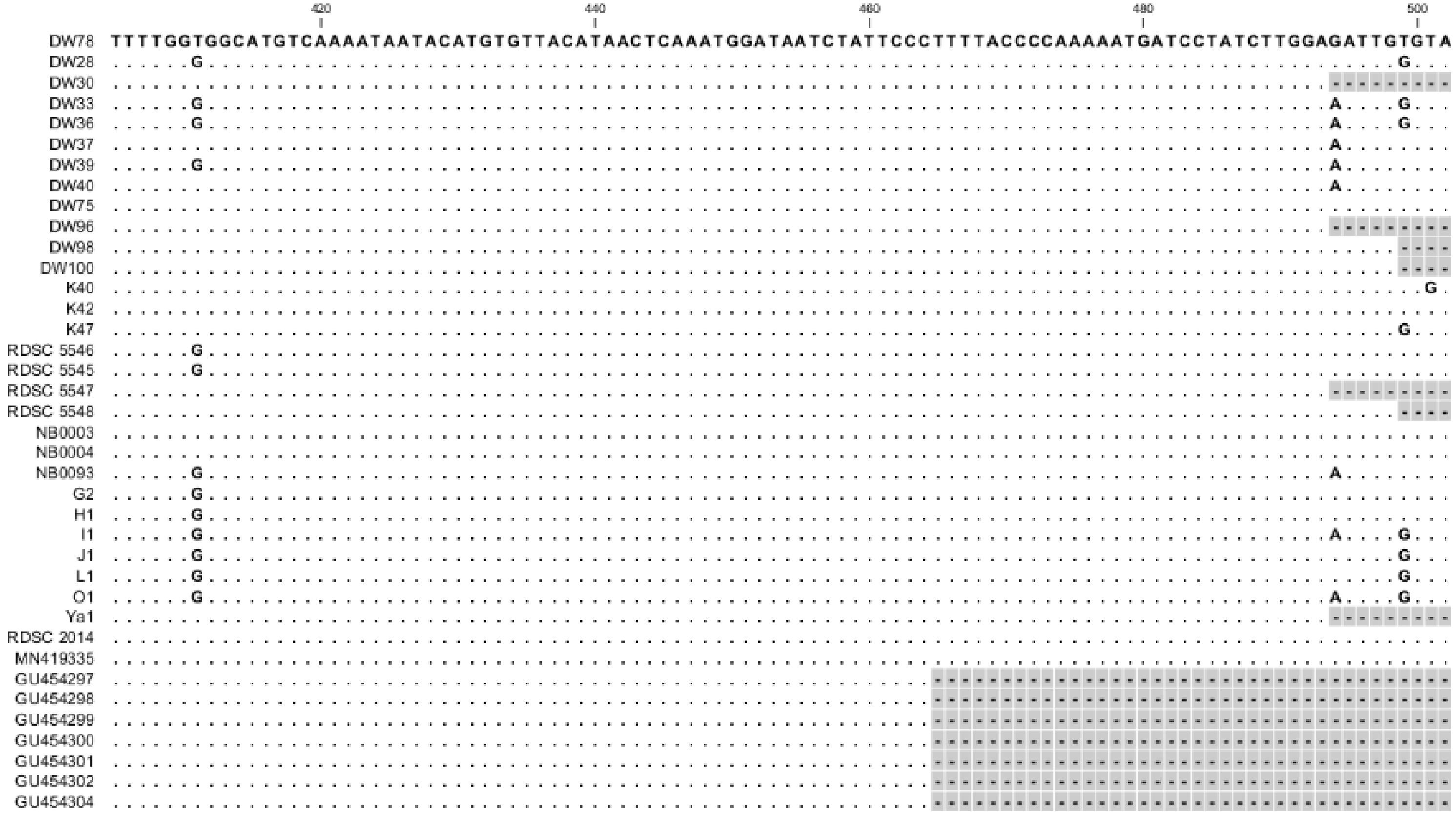

Supplemental Figure S1. Nucleotide alignment of *atpH–atpF* and *psbK–psbI* spacers sequences of *Spirodela polyrhiza* collected in Ukraine and China (for ecotype details see supplemental Table 2). Matching residues are shown as dots.

A. Nucleotide alignment of *atpH–atpF* barcodes including representative sequences of *S. polyrhiza* specimens available in GenBank: MN419335, USA; GU454201, Hong Kong; GU454202, India; GU454203, Malaysia; GU454204, USA; GU454205, Mexico; GU454206, Canada; GU454208, Europe.

B. Nucleotide alignment of *psbK-psbI* barcodes including representative sequences of *S. polyrhiza* specimens available in GenBank: MN419335, USA; GU454297, Hong Kong; GU454298, India; GU454299, Malaysia; GU454300, USA; GU454301, Mexico; GU454302, Canada; GU454304, Europe.

S2A

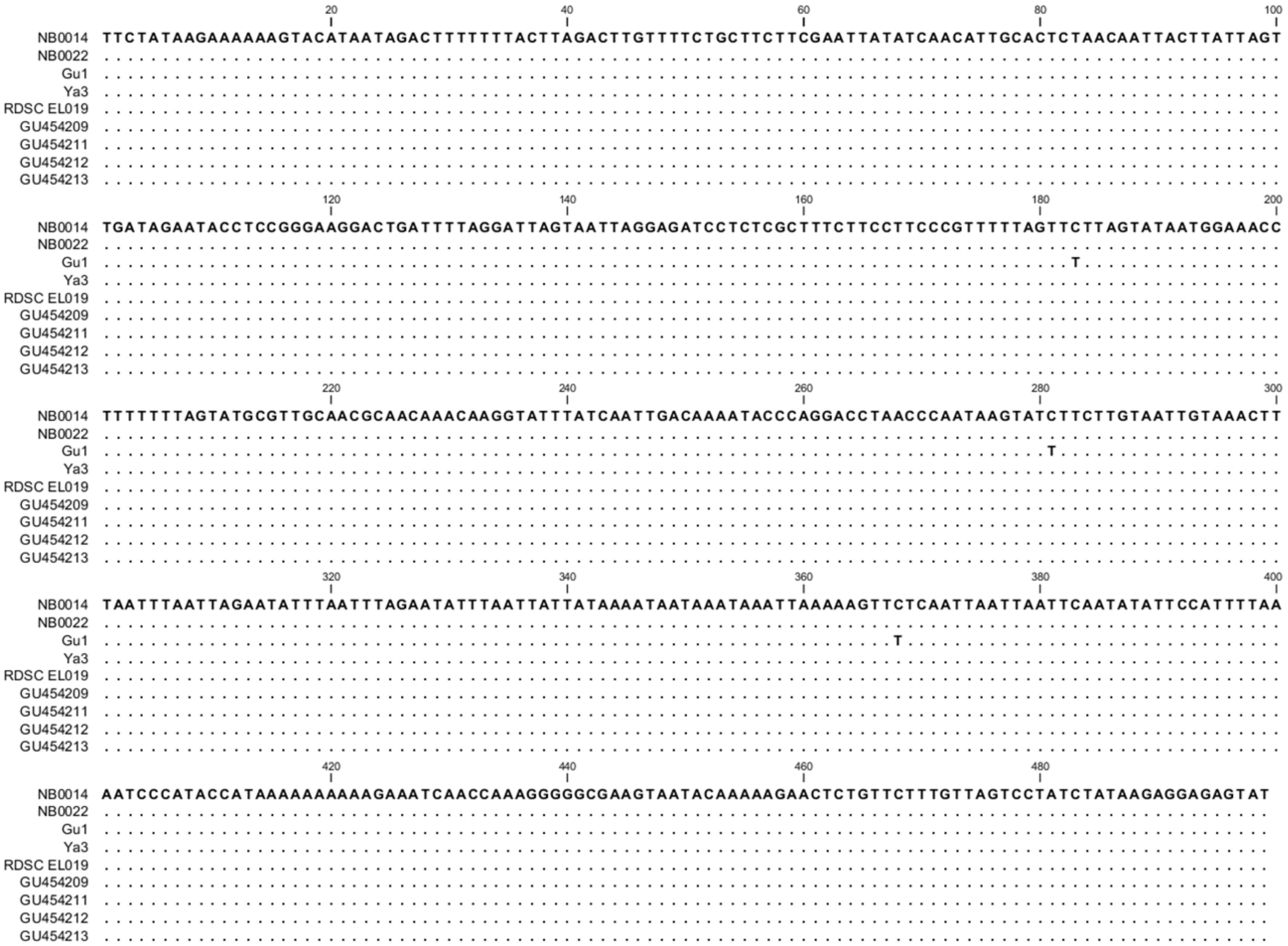
NB0014 TTCTATAAGAAAAAAGTACATAATAGACTTTTTTTACTTAGACTTGTTTTCTGCTTCTTCGAATTATATCAACATTGCACTCTAACAATTACTTATTAGT
NB0014 TGATAGAATACCTCCGGGAAGGACTGATTTTAGGATTAGTAATTAGGAGATCCTCTCGCTTTCTTCCTTCCCGTTTTTAGTTCTTAGTATAATGGAAACC
NB0014 TTTTTTTAGTATGCGTTGCAACGCAACAAACAAGGTATTTATCAATTGACAAAATACCCAGGACCTAACCCAATAAGTATCTTCTTGTAATTGTAAACTT
NB0014 TAATTTAATTAGAATATTTAATTTAGAATATTTAATTATTATAAAATAATAAATAAATTAAAAAGTTCTCAATTAATTAATTCAATATATTCCATTTTAA
NB0014 AATCCCATACCATAAAAAAAAAAAGAAATCAACCAAAGGGGGCGAAGTAATACAAAAAGAACTCTGTTCTTTGTTAGTCCTATCTATAAGAGGAGAGTAT
NB0022
Gu1
Ya3
RDSC EL019
GU454209
GU454211
GU454212
GU454213

S2B

```
                                    20                  40                  60                  80                 100
                                     |                   |                   |                   |                   |
NB0022     AGTTATTAAAACTCTATTGAAAAAATTCATGATTTATTTGATAAAAAAAGATTCTAATAAAGATAAGATAATATCAATTTTACGTTATACATCCTCATTC 100
NB0014     .................................................................................................... 100
Gu1        .................................................................................................... 100
Ya3        .................................................................................................... 100
RDSC EL019 .................................................................................................... 100
GU454305   .................................................................................................... 100
GU454307   .................................................................................................... 100
GU454308   .................................................................................................... 100
GU454309   .................................................................................................... 100

                                   120                 140                 160                 180                 200
                                     |                   |                   |                   |                   |
NB0022     CAAAATTTGGAATTCTTGTATATTGAATAAAAAGAGCGATAAGTTTTGATCAGTCCATTTCGGCCTTCTGGAGGCTCTTCCAGTGAGCAAGTACTTTATT 200
NB0014     .................................................................................................... 200
Gu1        .................................................................................................... 200
Ya3        .................................................................................................... 200
RDSC EL019 .................................................................................................... 200
GU454305   .................................................................................................... 200
GU454307   .................................................................................................... 200
GU454308   .................................................................................................... 200
GU454309   .................................................................................................... 200

                                   220                 240                 260                 280                 300
                                     |                   |                   |                   |                   |
NB0022     TATTAGCTTCTGTTTTCCACAATAATTTATTGAATATTAGAGTTAGTATTAGAAAAACCTTTTGGGTAACGAACAAATCATAATCTTAATTTCAACAACA 300
NB0014     .................................................................................................... 300
Gu1        .................................................................................................... 300
Ya3        ...................................................T................................................ 300
RDSC EL019 .................................................................................................... 300
GU454305   .................................................................................................... 300
GU454307   .................................................................................................... 300
GU454308   .................................................................................................... 300
GU454309   .................................................................................................... 300

                                   320                 340                 360                 380                 400
                                     |                   |                   |                   |                   |
NB0022     AAAAATTCATGAATTTTGTAATTCAGTTTTTTTAGAAAAAAACACTTAATAAAGAAAAAATTGTTCTTTATTTTTACTAGTTTTTTTTGTGGCATGTCAA 400
NB0014     .................................................................................................... 400
Gu1        .................................................................................................... 400
Ya3        .................................................................................................... 400
RDSC EL019 .................................................................................................... 400
GU454305   .................................................................................................... 400
GU454307   .................................................................................................... 400
GU454308   .................................................................................................... 400
GU454309   .................................................................................................... 400

                                   420                 440                 460                 480
                                     |                   |                   |                   |
NB0022     AATAAGACATGTGTTACATAACTCAAATGGATAATCTATTCCCTTTTACCCCAAAAATGATCCTATCTTGGAGATTGTGTA 481
NB0014     ..............................................................------------------- 462
Gu1        ................................................................................. 481
Ya3        ................................................................................. 481
RDSC EL019 ................................................................................. 481
GU454305   ...........................................-------------------------------------- 443
GU454307   ...........................................-------------------------------------- 443
GU454308   ...........................................-------------------------------------- 443
GU454309   ...........................................-------------------------------------- 443
```

Supplemental Figure S2. Nucleotide alignment of *atpH-atpF* and *psbK-psbI* spacers of *Landoltia punctata* (for ecotypes details see supplemental Table 2). Matching residues are shown as dots.

A. Nucleotide alignment of *atpH-atpF* barcodes including the representative sequences of *La. punctata* from different parts of the world available in the GenBank: GU454209, South Africa; GU454211, India; GU454212, USA; GU454213, Australia.

B. Nucleotide alignment of *psbK-psbI* barcodes including the representative sequences of *La. punctata* from different parts of the world available in the GenBank: GU454305, South Africa; GU454307, India; GU454308, USA; GU454309, Australia.

S3A

```
                                  20                  40                  60                  80                 100
                                   |                   |                   |                   |                   |
DW31      TTCTAGAATAAAAAAGTACGTAATAGACTTTTTGACTTAGACTTGCTTTTTGCTTCTTCGAATTATATCAACATTGTACTCTAACAATTACTTATTCGTT
DW38      ....................................................................................................
DW41      ....................................................................................................
DW72      ....................................................................................................
DW73      ....................................................................................................
DW74      ....................................................................................................
DW76      ....................................................................................................
DW99      ....................................................................................................
DW105     ....................................................................................................
K38       ....................................................................................................
K39       ....................................................................................................
K41       ....................................................................................................
K43       ....................................................................................................
K44       ....................................................................................................
K45       ....................................................................................................
K46       ....................................................................................................
K48       ....................................................................................................
Ya2       ....................................................................................................
DQ400350  ....................................................................................................
GU454226  ....................................................................................................
GU454227  ....................................................................................................
GU454228  ....................................................................................................
GU454229  ....................................................................................................
GU454230  ....................................................................................................
GU454231  ....................................................................................................

                                 120                 140                 160                 180                 200
                                   |                   |                   |                   |                   |
DW31      GAGAGAATACCTCCGGGAAGGACTGATTTTAGGATTAGTAATTAGCAGATCCTCTCGCTTTCTTCCTTCCCGTTTTCAGTTCTTAGTATAATGTAATGCA
DW38      ....................................................................................................
DW41      ....................................................................................................
DW72      ....................................................................................................
DW73      ....................................................................................................
DW74      ....................................................................................................
DW76      ....................................................................................................
DW99      ....................................................................................................
DW105     ....................................................................................................
K38       ....................................................................................................
K39       ....................................................................................................
K41       ....................................................................................................
K43       ....................................................................................................
K44       ....................................................................................................
K45       ....................................................................................................
K46       ....................................................................................................
K48       ....................................................................................................
Ya2       ....................................................................................................
DQ400350  ....................................................................................................
GU454226  ....................................................................................................
GU454227  ....................................................................................................
GU454228  ....................................................................................................
GU454229  ....................................................................................................
GU454230  ....................................................................................................
GU454231  ....................................................................................................
```

S3A

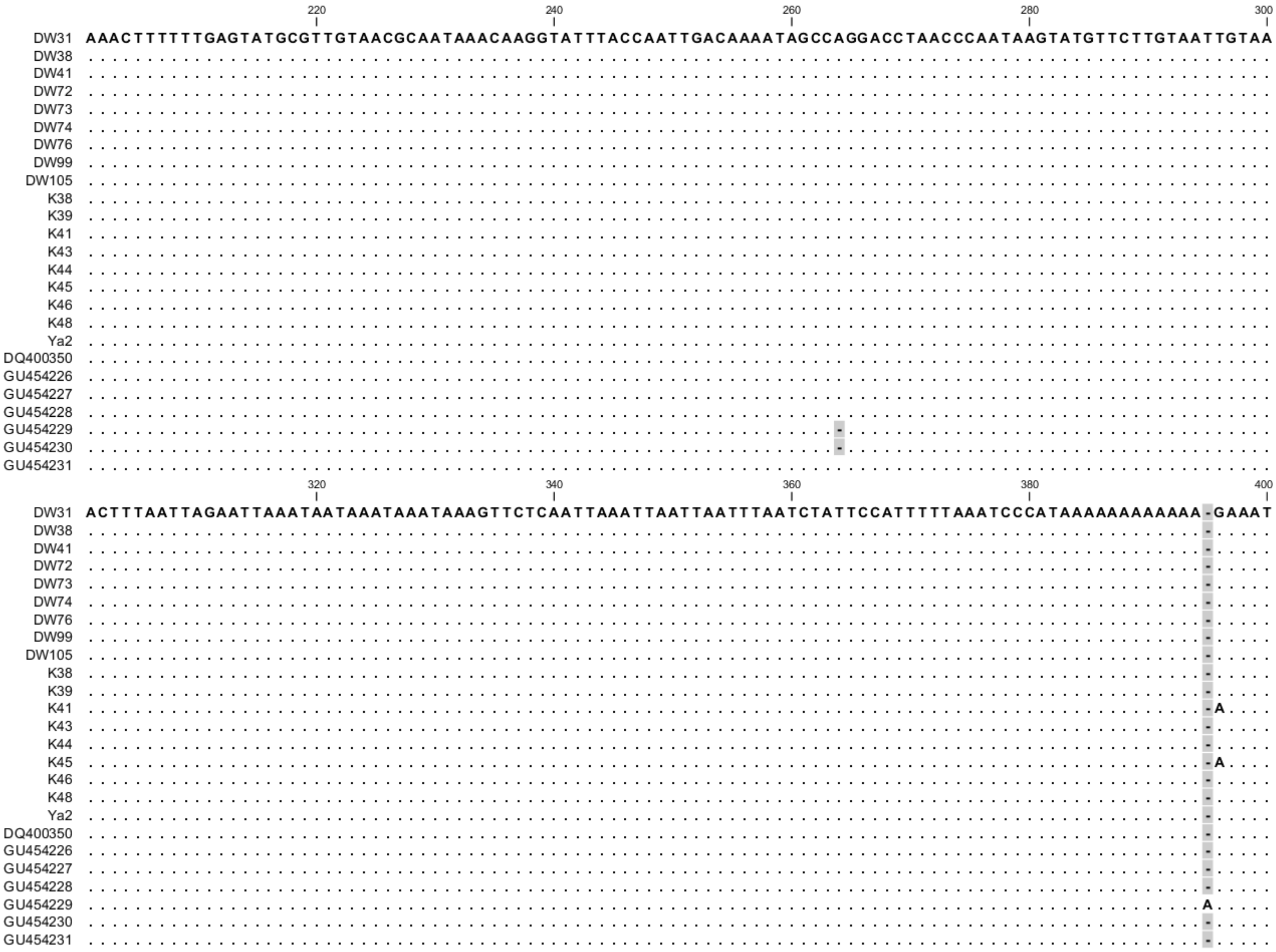
220
240
260
280
300
DW31 AAACTTTTTTGAGTATGCGTTGTAACGCAATAAACAAGGTATTTACCAATTGACAAAATAGCCAGGACCTAACCCAATAAGTATGTTCTTGTAATTGTAA
DW38
DW41
DW72
DW73
DW74
DW76
DW99
DW105
K38
K39
K41
K43
K44
K45
K46
K48
Ya2
DQ400350
GU454226
GU454227
GU454228
GU454229
GU454230
GU454231
320
340
360
380
400
DW31 ACTTTAATTAGAATTAAATAATAAATAAATAAAGTTCTCAATTAAATTAATTAATTTAATCTATTCCATTTTTAAATCCCATAAAAAAAAAAAA-GAAAT
DW38
DW41
DW72
DW73
DW74
DW76
DW99
DW105
K38
K39
K41
K43
K44
K45
K46
K48
Ya2
DQ400350
GU454226
GU454227
GU454228
GU454229
GU454230
GU454231

S3A

```
                 420                 440                 460
                   |                   |                   |
DW31      CAAACAAAGGGGGCGAAGTAATACAAAAGGAACTCTGTTCTTTTTTAGTCCTATCTATAAGAGGAGAGTAT 470
DW38      ....................................................................... 470
DW41      ....................................................................... 470
DW72      ....................................................................... 470
DW73      ....................................................................... 470
DW74      ....................................................................... 470
DW76      ....................................................................... 470
DW99      ....................................................................... 470
DW105     ....................................................................... 470
K38       ....................................................................... 470
K39       ............................................................A.......... 470
K41       ....................................................................... 470
K43       ....................................................................... 470
K44       ....................................................................... 470
K45       ............................................................A.......... 470
K46       ....................................................................... 470
K48       ..................G.........................................A.......... 470
Ya2       ....................................................................... 470
DQ400350  ....................................................................... 470
GU454226  ....................................................................... 470
GU454227  ....................................................................... 470
GU454228  ....................................................................... 470
GU454229  ....................................................................... 470
GU454230  ....................................................................... 469
GU454231  ....................................................................... 470
```

S3B

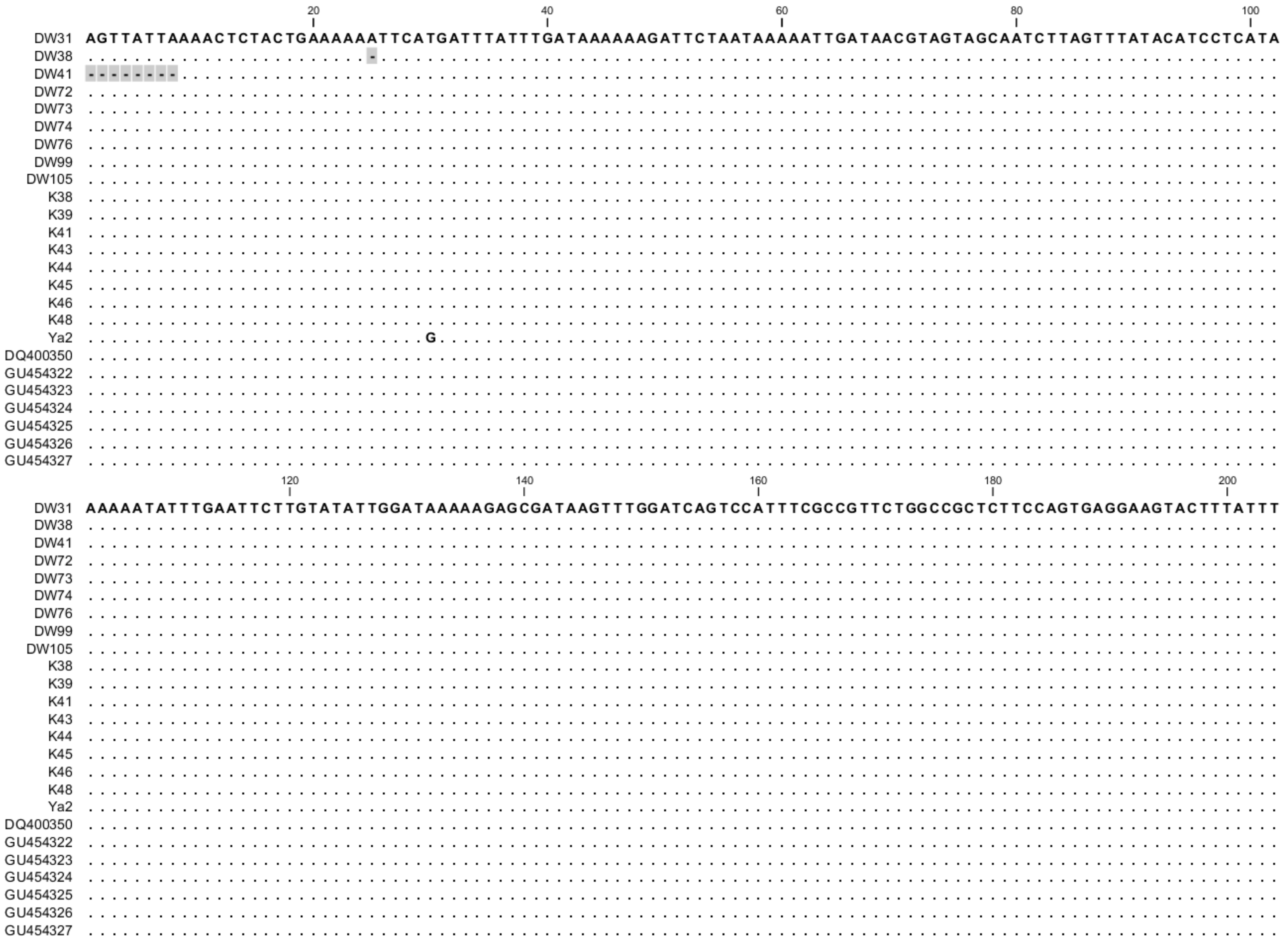
20
40
60
80
100
DW31 AGTTATTAAAACTCTACTGAAAAAATTCATGATTTATTTGATAAAAAAGATTCTAATAAAAATTGATAACGTAGTAGCAATCTTAGTTTATACATCCTCATA
DW38
DW41 --------
DW72
DW73
DW74
DW76
DW99
DW105
K38
K39
K41
K43
K44
K45
K46
K48
Ya2 G
DQ400350
GU454322
GU454323
GU454324
GU454325
GU454326
GU454327
120
140
160
180
200
DW31 AAAAATATTTGAATTCTTGTATATTGGATAAAAAGAGCGATAAGTTTGGATCAGTCCATTTCGCCGTTCTGGCCGCTCTTCCAGTGAGGAAGTACTTTATTT
DW38
DW41
DW72
DW73
DW74
DW76
DW99
DW105
K38
K39
K41
K43
K44
K45
K46
K48
Ya2
DQ400350
GU454322
GU454323
GU454324
GU454325
GU454326
GU454327

S3B

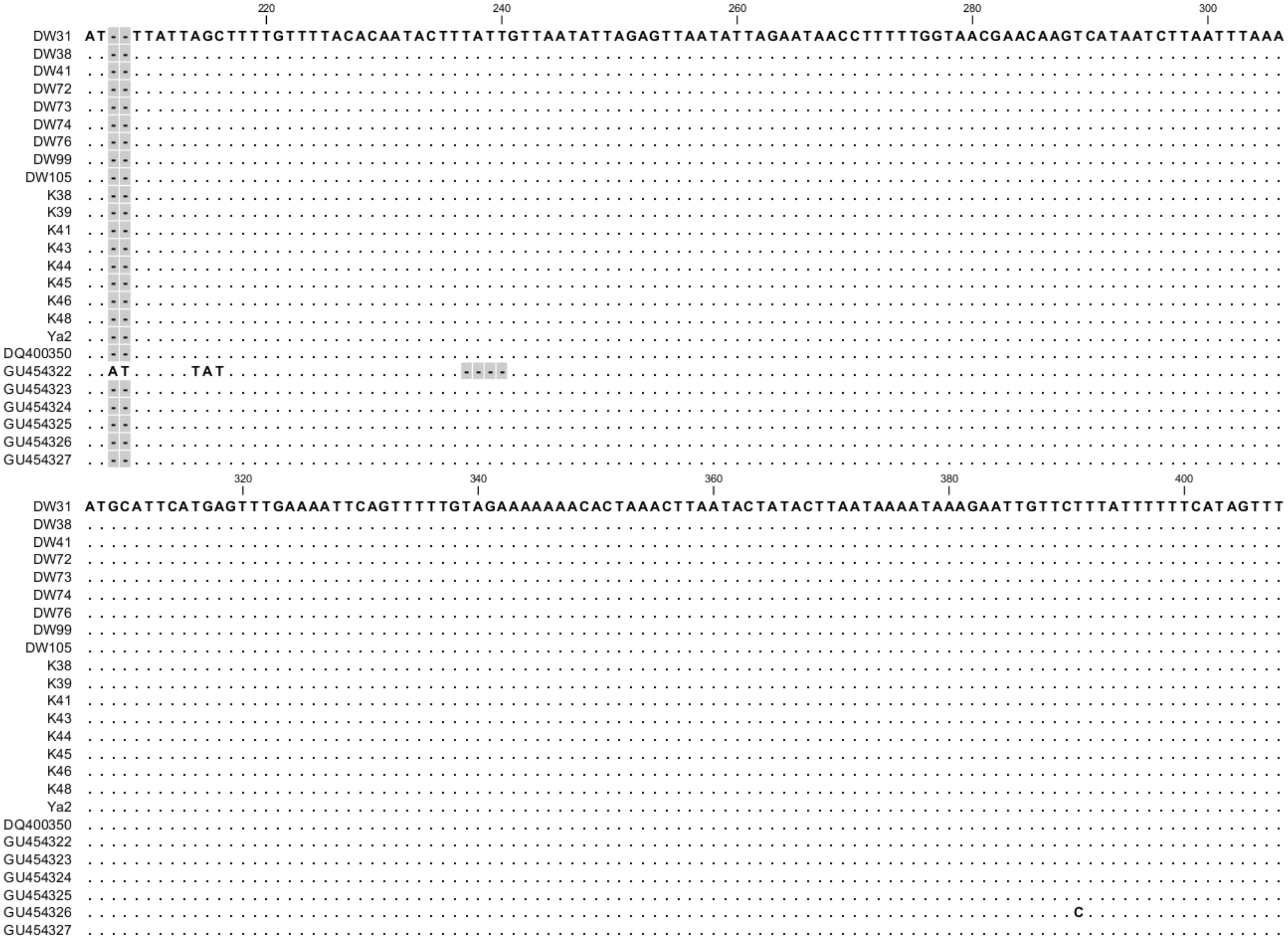
220
240
260
280
300
DW31 AT--TTATTAGCTTTTGTTTTACACAATACTTTATTGTTAATATTAGAGTTAATATTAGAATAACCTTTTTGGTAACGAACAAGTCATAATCTTAATTTAAA
DW38
DW41
DW72
DW73
DW74
DW76
DW99
DW105
K38
K39
K41
K43
K44
K45
K46
K48
Ya2
DQ400350
GU454322 AT TAT ----
GU454323
GU454324
GU454325
GU454326
GU454327
320
340
360
380
400
DW31 ATGCATTCATGAGTTTGAAAATTCAGTTTTTGTAGAAAAAAACACTAAACTTAATACTATACTTAATAAAATAAAGAATTGTTCTTTATTTTTTCATAGTTT
DW38
DW41
DW72
DW73
DW74
DW76
DW99
DW105
K38
K39
K41
K43
K44
K45
K46
K48
Ya2
DQ400350
GU454322
GU454323
GU454324
GU454325
GU454326 C
GU454327

S3B

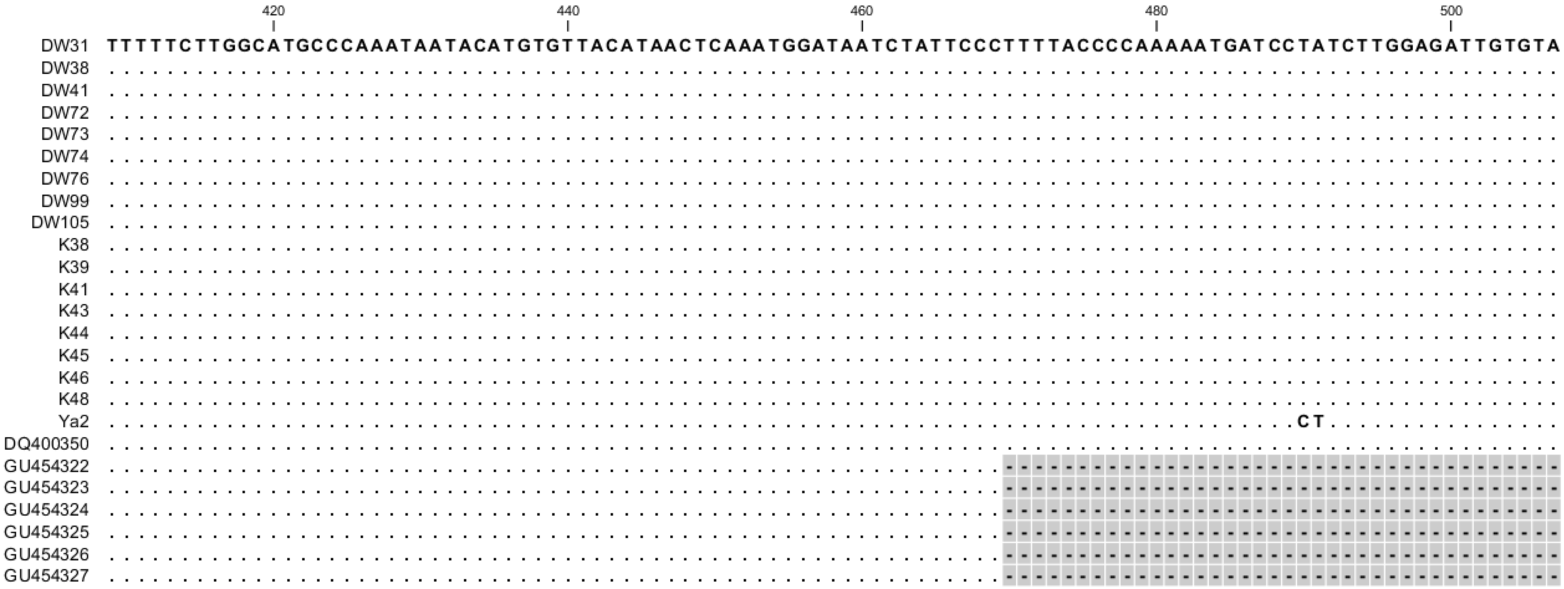

Supplemental Figure S3. Nucleotide alignment of *atpH-atpF* and *psbK-psbI* spacers of *Lemna minor* (for ecotypes details see supplemental Table 2). Matching residues are shown as dots.

A. Nucleotide alignment of *atpH-atpF* barcodes including the representative sequences of *Le. minor* from different parts of the world available in the GenBank: DQ400350, Russia; GU454226, Turkey; GU454227, USA; GU454228, South Africa; GU454229, Japan; GU454230, Finland; GU454231, Germany.

B. Nucleotide alignment of *psbK-psbI* barcodes including the representative sequences of *Le. minor* from different parts of the world available in the GenBank: DQ400350, Russia; GU454322, Turkey; GU454323, USA; GU454324, South Africa; GU454325, Japan; GU454326, Finland; GU454327, Germany.

S4A

```
                             20                  40                  60                  80                 100
DW34      TTCTAGAATAAAAAAGTACGTCATAGACTTTTTGACTTAGACTTGCTTTTTGCTTCTTCGAATTATATCAAGATTGTACTCTAACAATTACTTATTCGTT
DW95      ....................................................................................................
DW97      ....................................................................................................
K49       ....................................................................................................
GU454236  ....................................................................................................
GU454237  ....................................................................................................
GU454238  ....................................................................................................

                            120                 140                 160                 180                 200
DW34      GAGAGAATACCTCCGGGAAGGACTGATTTTAGGATTAGTAATTAGCAGATCCTCTCGCTTTCTTCCTTCCCGTTTTCAGTTCTTAGTATAATGTAATGCA
DW95      ....................................................................................................
DW97      ....................................................................................................
K49       ....................................................................................................
GU454236  ....................................................................................................
GU454237  ....................................................................................................
GU454238  ....................................................................................................

                            220                 240                 260                 280                 300
DW34      AAACTTTTTTGAGTATGCGTTGTAACGCAACAAACAAAGTATTTATCAATTGACAAAATAGCCAGGACCTAACCCAATAAGTATGTTCTTGTAATTGTAA
DW95      ....................................................................................................
DW97      ....................................................................................................
K49       ....................................................................................................
GU454236  ....................................................................................................
GU454237  ....................................................................................................
GU454238  ....................................................................................................

                            320                 340                 360                 380                 400
DW34      ACTTTAATTAGAATTAAATAATAAATAAAAAAGTTCTCAATTAAATTAATTAATTTAATCTATTCCATTTTAAAATCCCATAAAAAAAAGAAATCAAACA
DW95      ....................................................................................................
DW97      ....................................................................................................
K49       ....................................................................................................
GU454236  ....................................................................................................
GU454237  ....................................................................................................
GU454238  ....................................................................................................

                            420                 440                 460
DW34      AA----GGGGGCGAAGTAATACAAAAAGAACTCTGTTCTTTTTTAGTCCTATCTATAAGAGGAGAGTAT
DW95      ..----.................................................................
DW97      ..----.................................................................
K49       ..----.................................................................
GU454236  ..----.................................................................
GU454237  ..----.................................................................
GU454238  ..CAAA.................................................................
```

S4B

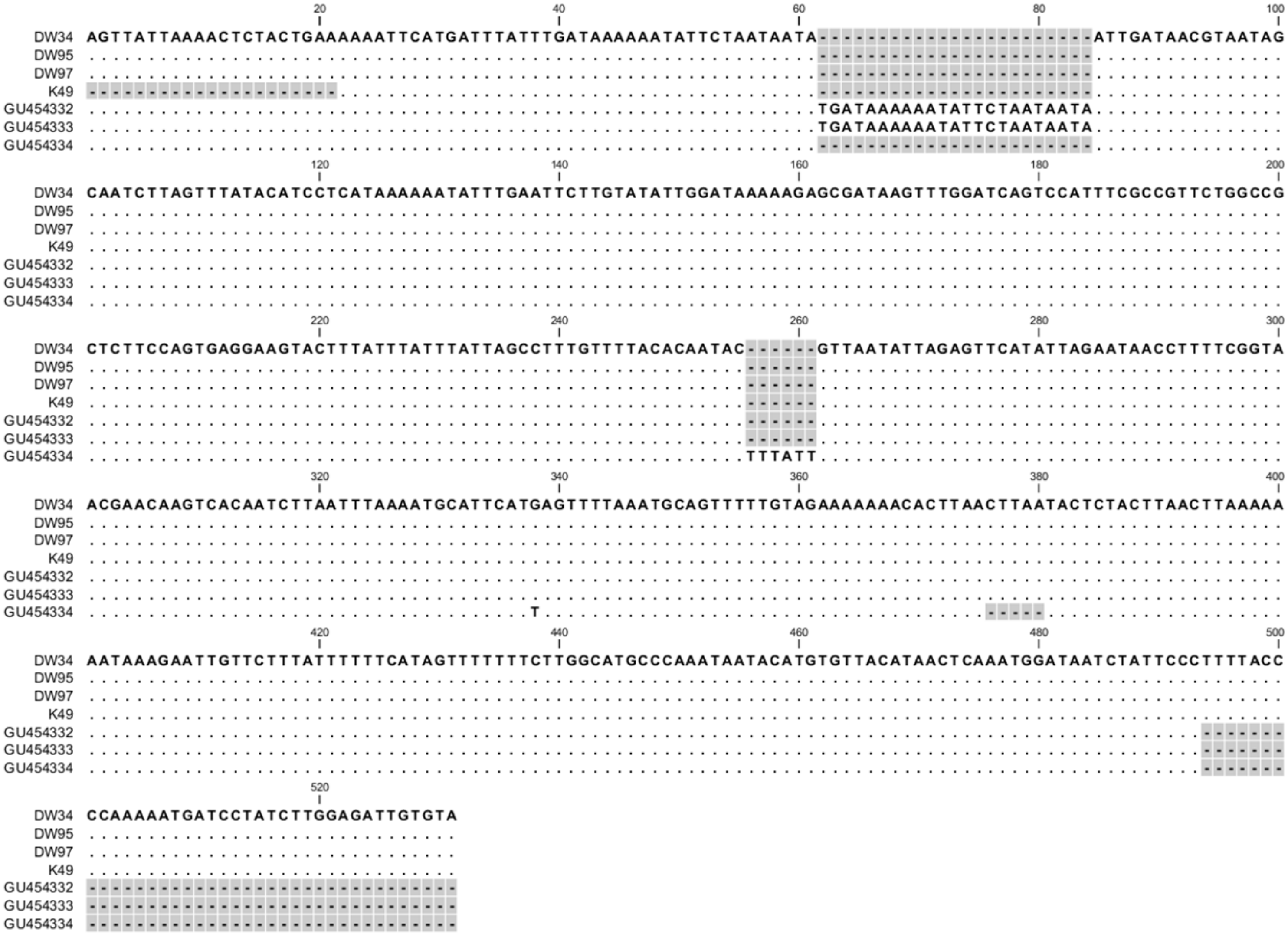

Supplemental Figure S4. Nucleotide alignment of *atpH-atpF* and *psbK-psbI* spacers of *Lemna trisulca* (for ecotypes details see supplemental Table 2). Matching residues are shown as dots.

A. Nucleotide alignment of *atpH-atpF* barcodes including the representative sequences of *Le. trisulca* from different parts of the world available in the GenBank: GU454236, Canada; GU454237, USA; GU454237, UTCC 399.

B. Nucleotide alignment of *psbK-psbI* barcodes including the representative sequences of *Le. trisulca* from different parts of the world available in the GenBank: GU454332, Canada; GU454333, USA; GU454334, UTCC 399.

S5A

```
                                     20                  40                  60                  80                 100
DW101     TTCTAGAATAAAAAAGTACGTAATAGACTTTTTGACTTAGACTTGCTTTTTGCTTCTTCGAATTATATCAAGATTGTACTCTAACAATTACTTATTCGTT
NB0013    ....................................................................................................
P2        ....................................................................................................
KF726146  ....................................................................................................
MG000422  ....................................................................................................
GU454240  ....................................................................................................
                                    120                 140                 160                 180                 200
DW101     GAGAGAATACCTCCGGGAAGGACTGATTTTAGGATTAGTAATTAGCAGATCCTCTCGCTTTCTTCCTTCCCGTTTTCAGTTCTTAGTATAATGTAATGCA
NB0013    ....................................................................................................
P2        ....................................................................................................
KF726146  ....................................................................................................
MG000422  ....................................................................................................
GU454240  ....................................................................................................
                                    220                 240                 260                 280                 300
DW101     AAACTTTTTTTGAGTATGCGTTGTAACGCAACAAACAAGGTATTTATCAATTGACAAAATAGCCAGGATCTAACCCAATAAGTATGTTCTTGTAATTATA
NB0013    ....................................................................................................
P2        ....................................................................................................
KF726146  ....................................................................................................
MG000422  ....................................................................................................
GU454240  ....................................................................................................
                                    320                 340                 360                 380                 400
DW101     AACTTTAATTAGAATTAAATAATAAATAAAAAAGTTCTCAATTAAATTAATTAATTAAATCTATTCCATTTAAAAATCCCATAAAAAAAAGAAATCAAAC
NB0013    ....................................................................................................
P2        ....................................................................................................
KF726146  ....................................................................................................
MG000422  ....................................................................................................
GU454240  ....................................................................................................
                                    420                 440                 460
DW101     AAAGGGGGCGAAGTAATACAAAAAGAACTCTGTTCTTTTTTAGTCCTATCTATAAGAGGAGAGTAT
NB0013    ..................................................................
P2        ..................................................................
KF726146  ..................................................................
MG000422  ..................................................................
GU454240  ..................................................................
```

S5B

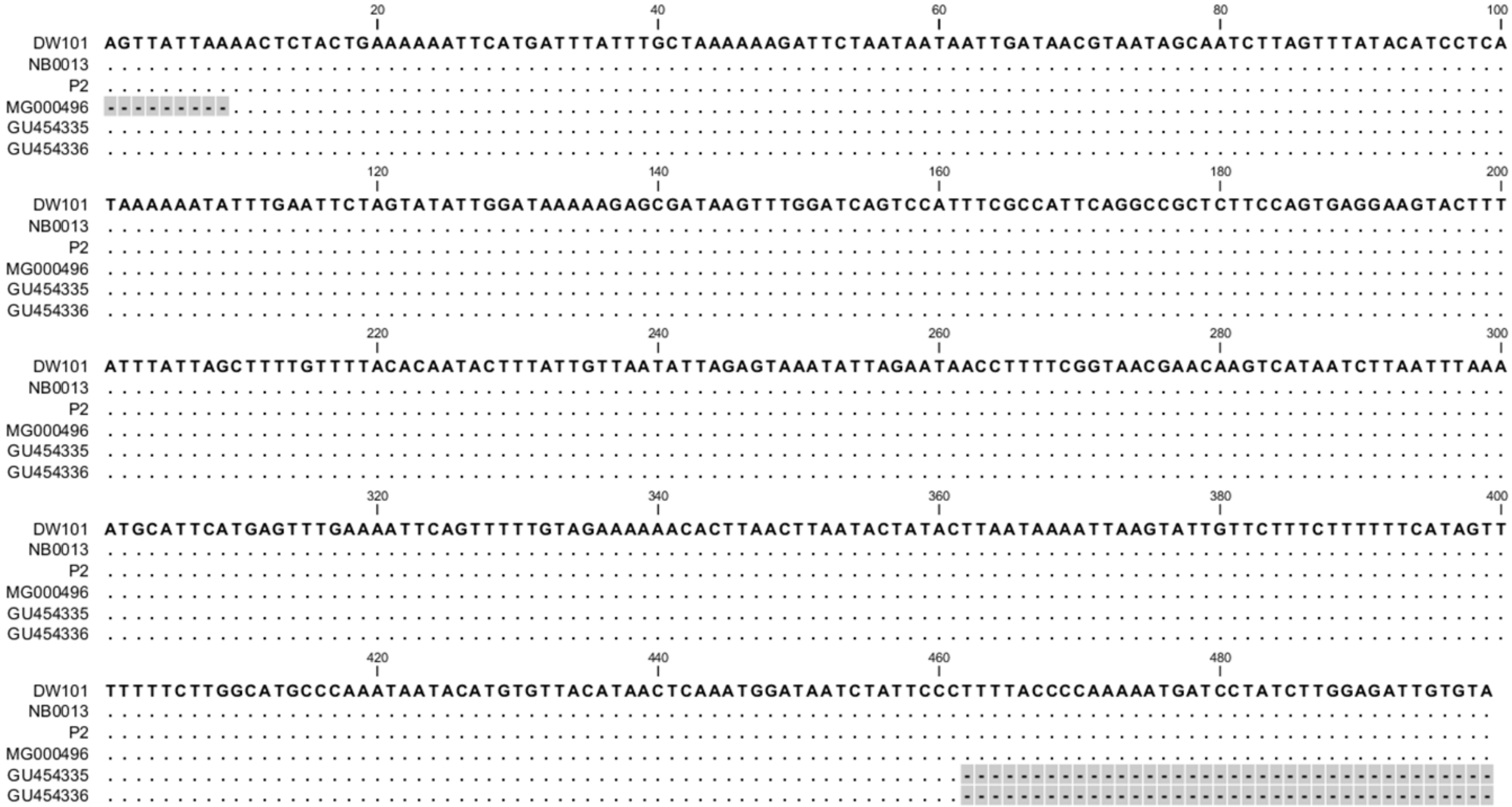

Supplemental Figure S5. Nucleotide alignment of *atpH-atpF* and *psbK-psbI* spacers of *Lemna turionifera* (for ecotypes details see supplemental Table 2). Matching residues are shown as dots.

A. Nucleotide alignment of *atpH-atpF* barcodes including the representative sequences of *Le. turionifera* from different parts of the world available in the GenBank: KF726146, China; MG000422, Canada; GU454240, Czech.

B. Nucleotide alignment of *psbK-psbI* barcodes including the representative sequences of *Le. turionifera* from different parts of the world available in the GenBank: MG000496, Canada; GU454335, China; GU454336, Czech.

S6A

```
                                 20                  40                  60                  80                 100
                                  |                   |                   |                   |                   |
DW102      TTTTAGAATCAAAAAAAGTACGTAATAGACTTTTTGACTTAGACTTGCTTTTTGCTTCTTCGAATTATATCAACATTGTACTCTAACAATTACTTATTCG
KX212889   ..C.................................................................................................
GU454219   ..C.................................................................................................
GU454220   ..C.................................................................................................
GU454221   ..C.................................................................................................
GU454222   ..C.................................................................................................

                                120                 140                 160                 180                 200
                                  |                   |                   |                   |                   |
DW102      TTGAGAGAATACCTCCGGGAAGGACTGATTTTAGGATTAGTAATTAGCAGATCCTCTCGCTTTCTTCCTTCCCGTTTTCAGTTCTTAGTATAATGTAATG
KX212889   ....................................................................................................
GU454219   ....................................................................................................
GU454220   ....................................................................................................
GU454221   ....................................................................................................
GU454222   ....................................................................................................

                                220                 240                 260                 280                 300
                                  |                   |                   |                   |                   |
DW102      CAAAATTTTTTAGAGTATGCGTTGTAACGCAATAAACAAGGTATTTACCAATTGACAAAATAGCCAGGACCTAACCCAATAAGTATGTTCTTGTAATTGT
KX212889   ....................................................................................................
GU454219   ....................................................................................................
GU454220   ....................................................................................................
GU454221   ....................................................................................................
GU454222   ....................................................................................................

                                320                 340                 360                 380                 400
                                  |                   |                   |                   |                   |
DW102      AAACTTTAATTAGAATTAAATAATAAATAAAAAAGTTCTCAATTAAATTAATTAATTTAATCTATTCCATTTTAAAATCCCATAAAAAAAAAGAAATCAA
KX212889   ....................................................................................................
GU454219   ....................................................................................................
GU454220   ....................................................................................................
GU454221   ....................................................................................................
GU454222   ....................................................................................................

                                420                 440                 460
                                  |                   |                   |
DW102      ACAAAGGGGGCGAAGTAATACAAAAAGAACAGAACTCTGTTCTTTTTTAGTCCTATCTATAAGAGGAGAGTAT
KX212889   .........................................................................
GU454219   .........................................................................
GU454220   .........................................................................
GU454221   .........................................................................
GU454222   .........................................................................
```

S6B

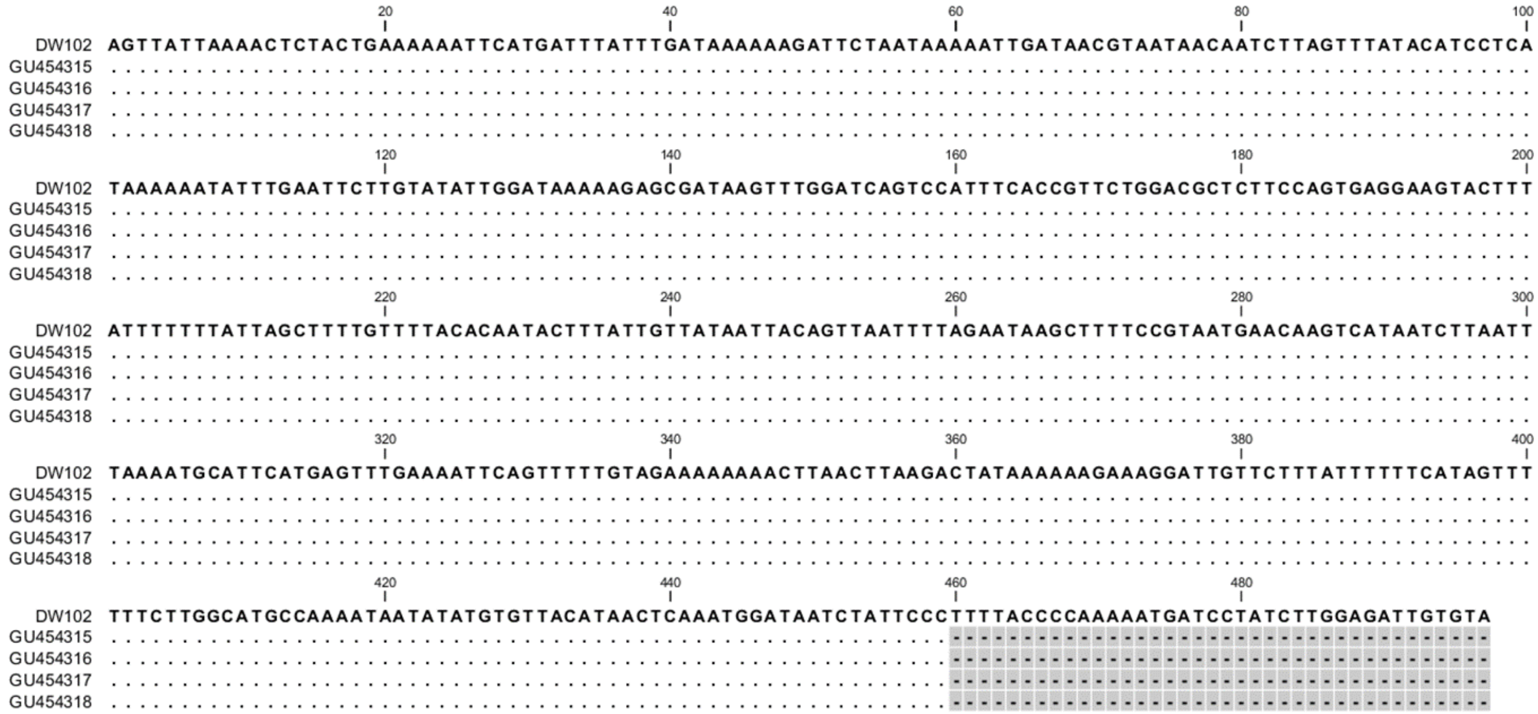

Supplemental Figure S6. Nucleotide alignment of *atpH-atpF* and *psbK-psbI* spacers of *Lemna gibba,* ecotype DW102 (Supplemental Table 2). Matching residues are shown as dots.

A. Nucleotide alignment of the DW102 *atpH-atpF* barcode with the representative sequences of *Le. gibba* from different parts of the world available in the GenBank: KX212889, China; GU454219, USA; GU454220, Italy; GU454221, Ethiopia; GU454222, Japan.

B. Nucleotide alignment of the DW102 *psbK-psbI* barcode with the representative sequences of *Le. gibba* from different parts of the world available in the GenBank: GU454315, USA; GU454316, Italy; GU454317, Ethiopia; GU454318, Japan.

S7A

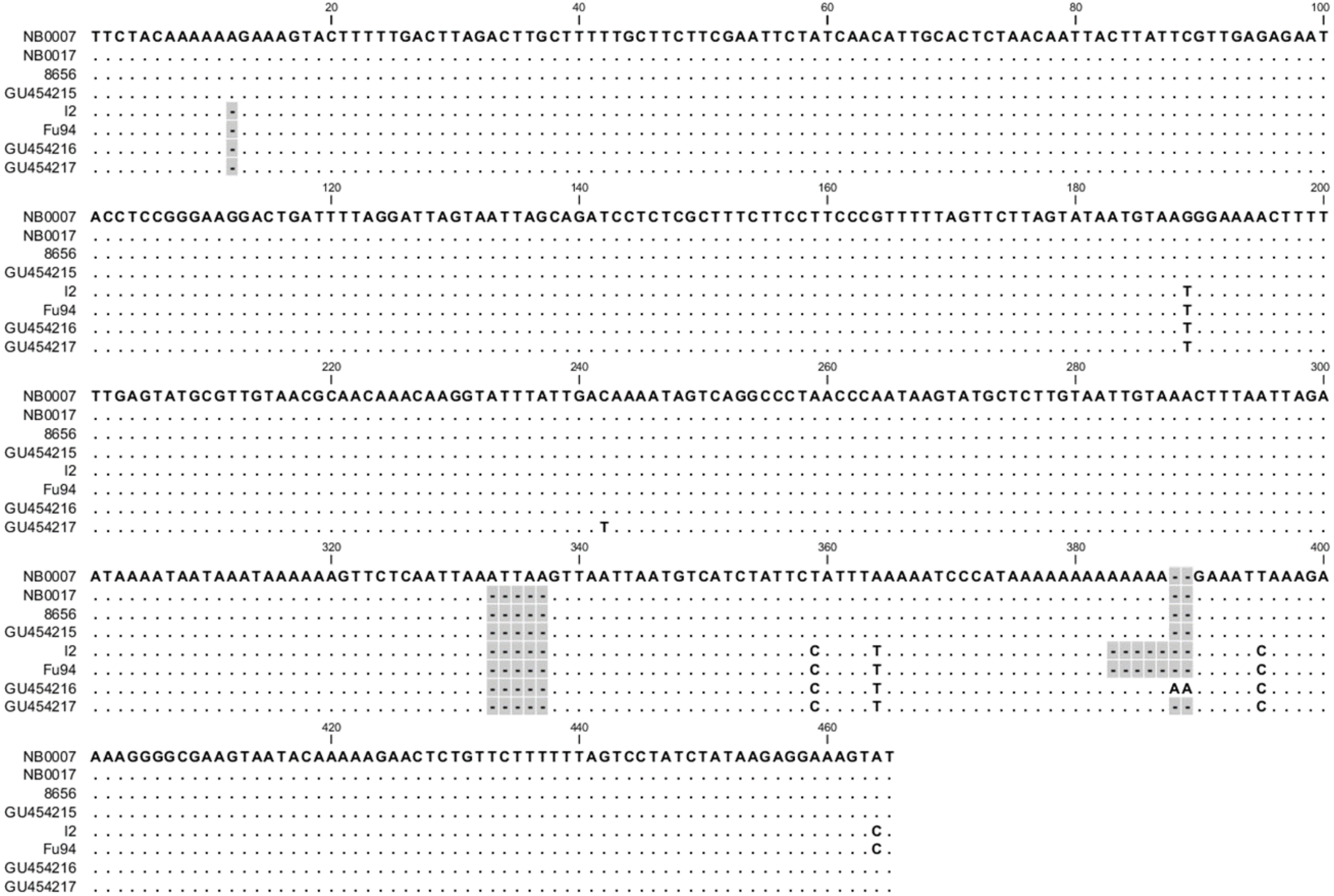

NB0007
NB0017
8656
GU454215
I2
Fu94
GU454216
GU454217
TTCTACAAAAAAGAAAGTACTTTTTGACTTAGACTTGCTTTTTGCTTCTTCGAATTCTATCAACATTGCACTCTAACAATTACTTATTCGTTGAGAGAAT
ACCTCCGGGAAGGACTGATTTTAGGATTAGTAATTAGCAGATCCTCTCGCTTTCTTCCTTCCCGTTTTTAGTTCTTAGTATAATGTAAGGGAAAACTTTT
TTGAGTATGCGTTGTAACGCAACAAACAAGGTATTTATTGACAAAATAGTCAGGCCCTAACCCAATAAGTATGCTCTTGTAATTGTAAACTTTAATTAGA
ATAAAATAATAAATAAAAAAGTTCTCAATTAAATTAAGTTAATTAATGTCATCTATTCTATTTAAAAATCCCATAAAAAAAAAAAAA--GAAATTAAAGA
AAAGGGGCGAAGTAATACAAAAAGAACTCTGTTCTTTTTTAGTCCTATCTATAAGAGGAAAGTAT

S7B

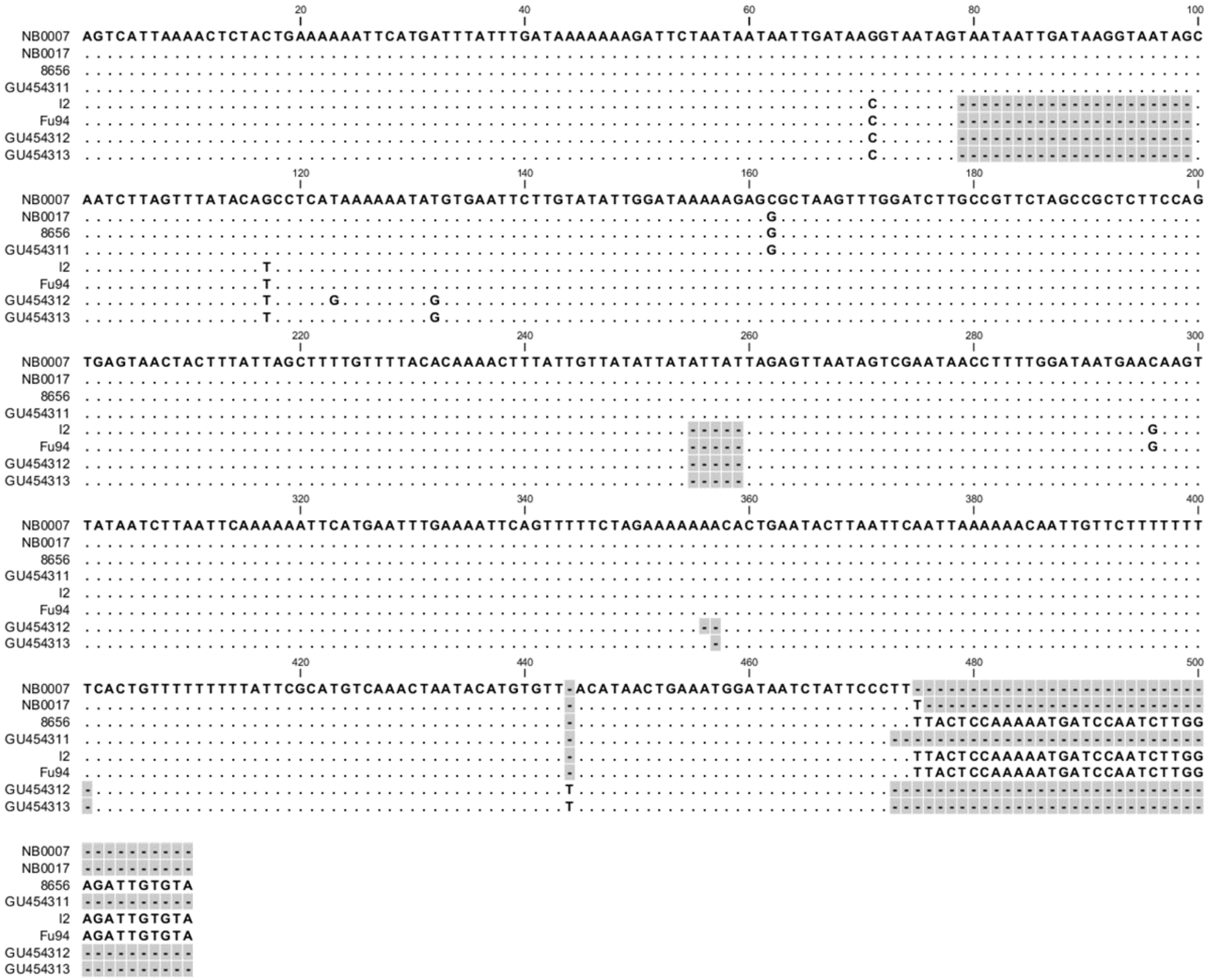

Supplemental Figure S7. Nucleotide alignment of *atpH-atpF* and *psbK-psbI* spacers of *Lemna aequinoctialis* (for ecotypes details see supplemental Table 2). Matching residues are shown as dots.

A. Nucleotide alignment of *atpH-atpF* barcodes including the representative sequences of *Le. aequinoctialis* from different parts of the world available in the GenBank: GU454215, USA, California, Centerville; GU454216, USA, California, Plainsburg; GU454217, USA, Texas, Austin.

B. *Nucleotide alignment of* psbK-psbI barcodes including the representative sequences of *Le. aequinoctialis* from different parts of the world available in the GenBank: GU454311, USA, California, Centerville; GU454312, USA, California, Plainsburg; GU454313, USA, Texas, Austin.

S8A

```
                            20                  40                  60                  80                 100
DW32      TTCTCGAATAAAAAAGTACGTCATAGTGTTTTTGACTTAGACTTGCTTTTTGCTTGTTCGAATTATCTCTAAATTGCACTATAACAATTACTTATTCGTT
DW35      ....................................................................................................
MG775412  ....................................................................................................
MG775413  ....................................................................................................
MG812317  ....................................................................................................

                           120                 140                 160                 180                 200
DW32      GAGAGAATACCTCCGGGAAGGACTGATTTTAGGATTAGTAATTAGCAGATCCTCTCGCTTTCTTCCTTCCCGTTTTTAGTTCGTAGTcTAATGGAAACCT
DW35      ....................................................................................................
MG775412  ....................................................................................................
MG775413  ....................................................................................................
MG812317  ....................................................................................................

                           220                 240                 260                 280                 300
DW32      TTTTTAGTATGCGTTGCAACGCAACAAACAAGGTAGTTATCAATTGACAAAATACCCAGGACCTAACCCAATAAGTAGATTCTTGTAATTGTAAACTTTA
DW35      ....................................................................................................
MG775412  ....................................................................................................
MG775413  ....................................................................................................
MG812317  ....................................................................................................

                           320                 340                 360                 380                 400
DW32      ATTTGTTTTTTTTTTATAAAAAAATAAAA--GTTTCTCAATTAAATTAATTAATCGATTAAAAATAAAAAATCCCACAAAAAAA-GAAATCAACCAAAGC
DW35      .............................--.....................................................-...............
MG775412  .............................--.....................................................-...............
MG775413  ...........A.AA.A.......A....AA.....................................................A...............
MG812317  .............................--.....................................................-...............

                           420                 440                 460
DW32      AAAGGGGGGG-AGTAATACAAAAAGAACTCTGTTCTTTGTTAGTCCTATCTATAAGAGGAGAGTAT
DW35      ..........-.......................................................
MG775412  ..........-.......................................................
MG775413  ..........G.......................................................
MG812317  ..........-.......................................................
```

S8B

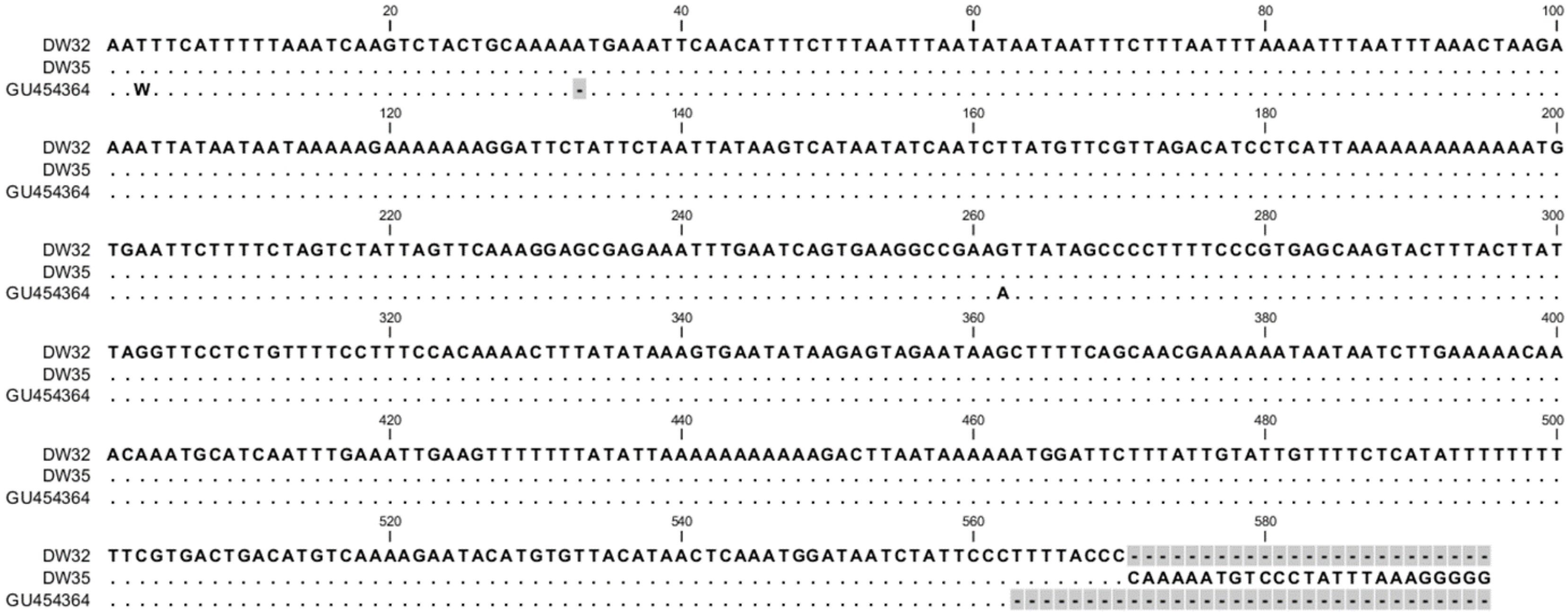

Supplemental Figure S8. Nucleotide alignment of *atpH-atpF* and *psbK-psbI* spacers of *Wolffia arrhiza* (for ecotypes details see supplemental Table 2). Matching residues are shown as dots.

A. Nucleotide alignment of *atpH-atpF* barcodes including the representative sequences of *W. arrhiza* from different parts of the world available in the GenBank: MG812317, Kenya; MG775412, Italy; MG775413, Brazil.

B. Nucleotide alignment of *psbK-psbI* barcodes including the representative sequence of *W. arrhiza* available in the GenBank: GU454364, Hungary.

S9A

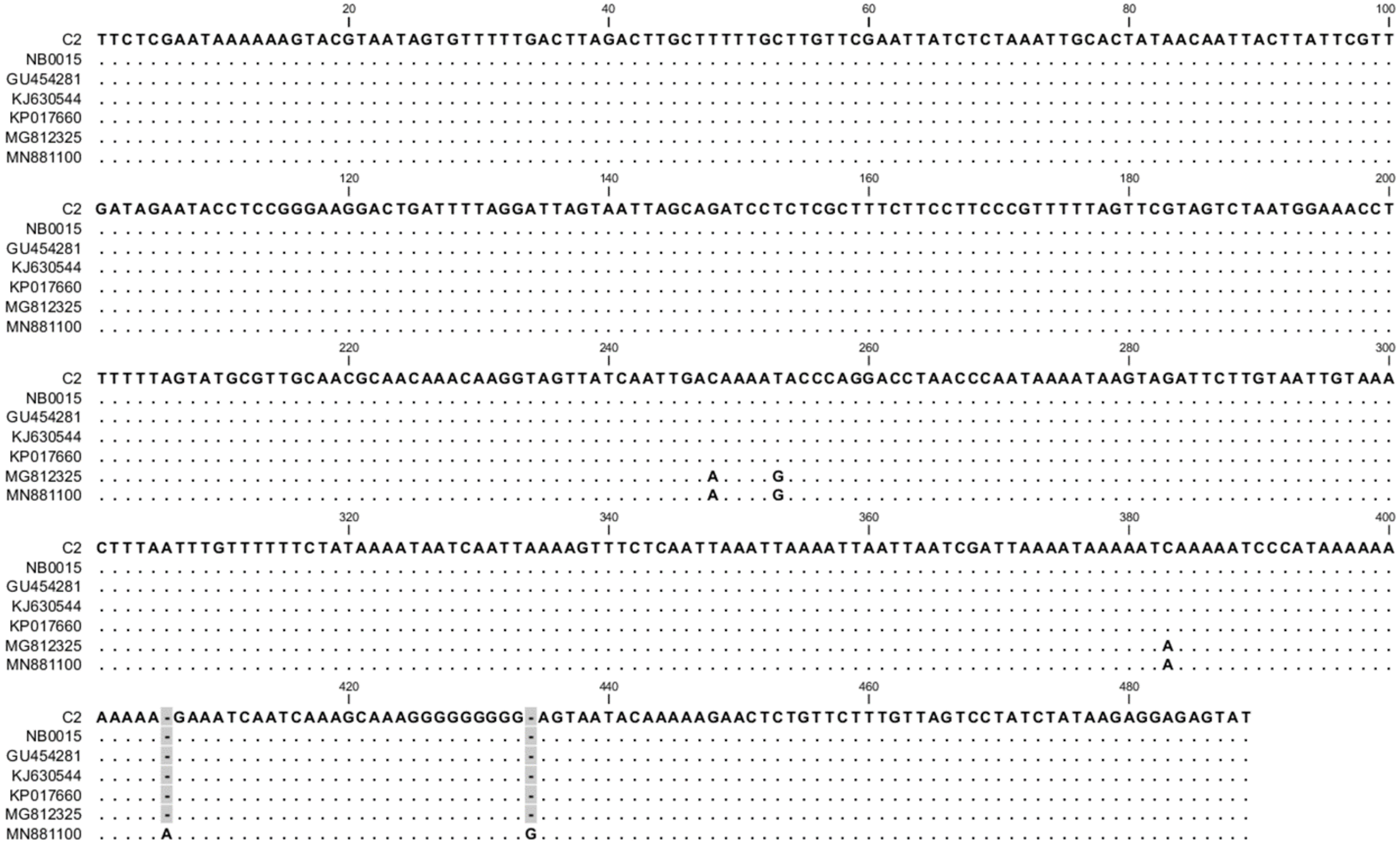
C2
NB0015
GU454281
KJ630544
KP017660
MG812325
MN881100
20 40 60 80 100
TTCTCGAATAAAAAAGTACGTAATAGTGTTTTTGACTTAGACTTGCTTTTTGCTTGTTCGAATTATCTCTAAATTGCACTATAACAATTACTTATTCGTT
120 140 160 180 200
GATAGAATACCTCCGGGAAGGACTGATTTTAGGATTAGTAATTAGCAGATCCTCTCGCTTTCTTCCTTCCCGTTTTTAGTTCGTAGTCTAATGGAAACCT
220 240 260 280 300
TTTTTAGTATGCGTTGCAACGCAACAAACAAGGTAGTTATCAATTGACAAAATACCCAGGACCTAACCCAATAAAATAAGTAGATTCTTGTAATTGTAAA
320 340 360 380 400
CTTTAATTTGTTTTTTCTATAAAATAATCAATTAAAAGTTTCTCAATTAAATTAAAATTAATTAATCGATTAAAATAAAAATCAAAAATCCCATAAAAAA
420 440 460 480
AAAAA-GAAATCAATCAAAGCAAAGGGGGGGGG-AGTAATACAAAAAGAACTCTGTTCTTTGTTAGTCCTATCTATAAGAGGAGAGTAT

S9B

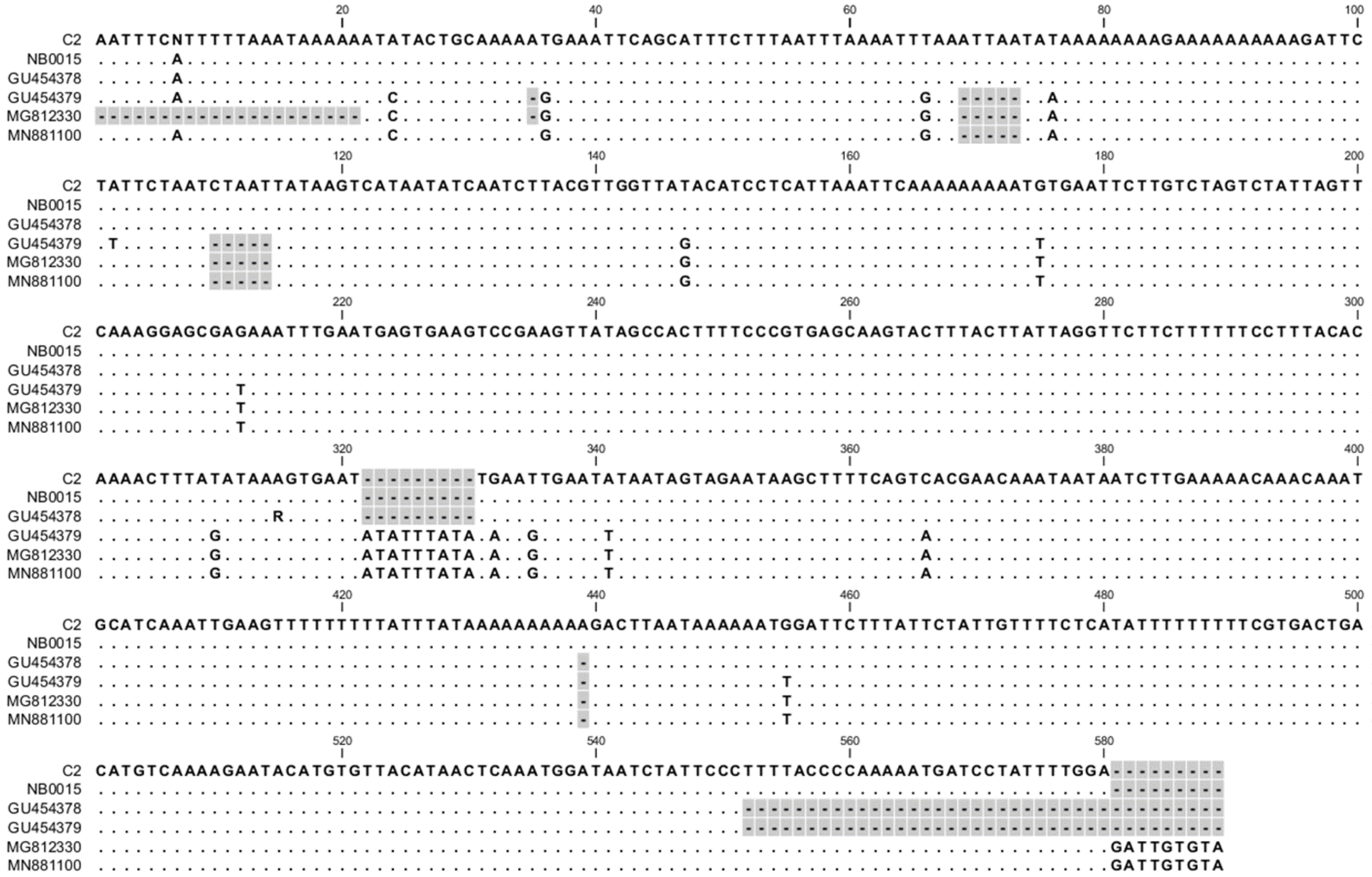

Supplemental Figure S9. Nucleotide alignment of *atpH-atpF* and *psbK-psbI* spacers of *Wolffia globosa* (for ecotypes details see supplemental Table 2). Matching residues are shown as dots.

A. Nucleotide alignment of *atpH-atpF* barcodes including the representative sequences of *W. globosa* from different parts of the world available in the GenBank: GU454281, USA; KJ630544, China; KP017660, China; MG812325, India; MN881100, Japan.

B. Nucleotide alignment of *psbK-psbI* barcodes including the representative sequences of *W. globosa* from different parts of the world available in the GenBank: GU454378, USA; GU454379, Thailand; MG812330, India; MN881100, Japan.
