# Supplementary material for: Biodiversity of Duckweed (Lemnaceae) in Water Reservoirs of Ukraine and China Assessed by Chloroplast DNA Barcoding
Source: Plants (Basel). 2022 May 30;11(11):1468. doi: 10.3390/plants11111468 (PMC9182681; doi:10.3390/plants11111468)
Supplement: Supplementary file 1 [file plants-11-01468-s001.zip › plants-1691397-SI/REVISED- Chen-Supplemental Tables.pdf]

Supplemental Table S1. The number of reference barcodes assigned to the duckweed species analyzed in this work that are publicly available in NCBI.Sequences obtained in this publication are marked by red.

Species	# strains analyzed with *psbK-psbI*	# strains analyzed with	References
Spirodela polyrhiza	10+30	92+30	[21, 35, 37, 39, 40, 41, 61, 62, 63]
Landoltia punctata	8+5	43+5	[39, 40, 41, 61, 62, 63, 64, 65]
Lemna gibba	8+1	12+1	[39, 66, 67]
Lemna minor	32+18	36+18	[34, 39, 40, 62, 66, 67, 68]
Lemna turionifera	70+3	75+3	[17, 39, 40, 62, 69]
Lemna trisulca	3+4	4+4	[39, 69]
Lemna aequinoctialis	4+4	103+4	[39, 40, 41, 61, 62, 64, 69]
Wolffia arrhiza	1+2	7+2	[39, 69, 70]
Wolffia globosa	14+2	31+2	[39, 41, 61, 62, 69, 70, 71]
**Total:**	**219+69**	**472+69**	
**Sum:**	**691+130**		

Supplemental Table S2. List of duckweed species genotyped in this study.

**Strain**	**Location of collection**	**GPS**	**AtpH-AtpF Acc #**	**PsbK-PsbI Acc #**
Spirodela polyrhiza				
DW28	Ukraine, Kyiv, Golosijiv Lake	50°22'44"N 30°30'58"E	OM569492	OM569553
DW30	Ukraine, Kyiv Oblast Tacenky	50°09'22"N 30°40'21"E	OM569493	OM569554
DW33	Ukraine, Kyiv Oblast,Kruhlyk	50°18'12"N 30°26'35"E	OM569494	OM569555
DW36	Ukraine, Kyiv Oblast,Kruhlyk	50°18'18"N 30°26'12"E	OM569495	OM569556
DW37	Ukraine, Kyiv Oblast,Kruhlyk	50°18'05"N 30°26'06"E	OM569496	OM569557
DW39	Ukraine, Kyiv Oblast, Maliutianka	50°17'25"N 30°16'20"E	OM569497	OM569558
DW40	Ukraine, Kyiv Oblast,Vita Poshtova	50°18'38"N 30°23'20"E	OM569498	OM569559
DW75	Ukraine, Rivne Oblast, Pasiky	50°16'36"N 25°25'58"E	OM793611	OM793616
DW78	Ukraine, Cherkasy Oblast, Luka, river Ros	49°34'34"N 31°26'51"E	OM569499	OM569560

DW96	Ukraine, Gluboke Lake (The Chernobyl Nuclear Power Plant Exclusion Zone**)**	51°26'39" N 30°03'54" E	OM569500	OM569561
DW98	Ukraine, Pripyat river (The Chernobyl Nuclear Power Plant Exclusion Zone)	51°16'36" N 30°14'28" E	OM569501	OM569562
DW100	Ukraine, Sumy Oblast, Lyntvarivka	50°58'58"N 34°36'57"E	OM569502	OM569563
K40	Ukraine, Kyiv Oblast, Borodyanka, Zdvyzh River	50°38'02"N 29°55'42"E	OM569503	OM569564
K42	Ukraine, Kyiv Oblast, Tal' River near Halynka	50°40'17"N 29°49'49"E	OM569504	OM569565
K47	Ukraine, Kyiv Oblast, Nemishajeve, local pond	50°34'21"N 30°05'26"E	OM569505	OM569566
RDSC 5545	China, Jiangsu, Huai'an	33°38′41"N 118°58′33"E	OM569506	OM569567
RDSC 5546	China, Jiangsu, channel near Hongze lake	33°22′43"N 118°53′23"E	OM569507	OM569568
RDSC 5547	China, Jiangsu, Hongze lake	33°19′41"N 118°51′43"E	OM569508	OM569569
RDSC 5548	China, Jiangsu, Hongze lake	33°17′40"N 118°49′45"E	MZ436185	MZ436186
NB0003	China, Jiangsu, Huai'an	33°35'09"N 119°01'27"E	OM569509	OM569570
NB0004	China, Jiangsu, Huai'an, Chinese academia of agriculture, rice field	33°28′59"N 119°2′1"E	OM569510	OM569571
NB0093	China,Fujian, Fuzhou	26°5'8"N 119°17'35"E	OM569511	OM569572
G2	China, Jiangsu, Huai'an	33°38′41"N 118°58′33"E	OM569512	OM569573
H1	China,Jiangsu, Huai'an	33°38'41" N 118°58'33" E	OM569513	OM569574
I1	China,Jiangsu, Huai'an	33°38'41"N 119°00'15"E	OM569514	OM569575
J1	China,Jiangsu, Huai'an	33°29'17"N 118°58'29"E	OM569515	OM569576
L1	China,Jiangsu, Huai'an	33°35'52"N 119°03'09"E	OM569516	OM569577
O1	China,Jiangsu, Huai'an	33°31'12"N 119°08'21"E	OM569517	OM569578
Ya1	China, Shaanxi, Yangling	34°18'06"N 108°02'22"E	OM569518	OM569579
RDSC 2014	China, Yunnan, Kunming	-	OM569519	OM569580
Landoltia punctata				
NB0014	China,Jiangsu, Huai'an	33°37'07"N 119°00'07"E	OM569520	OM569581
NB0022	China,Jiangsu, Huai'an	33°38'33"N	OM569521	OM569582

		119°00'38"E		
Gu1	China, Guangxi Zhuang Autonomous Region, Guilin	25°16'15"N 110°17'44"E	OM569522	OM569583
Ya3	China, Shaanxi, Yangling	34°18'06"N 108°02'22"E	OM569523	OM569584
RDSC EL019	China, Yunnan, Kunming	-	OM569524	OM569585
Lemna aequinoctialis				
I2	China, Jiangsu, Huai’an	33°38'41"N 119°00'15"E	OM569525	OM569586
NB0017	China, Jiangsu, Huai’an	33°37'07"N 119°00'07"E	OM569526	OM569587
NB0007	China, Shanghai	31°13'52"N 121°28'19"E	MZ436181	MZ436182
Fu94	China, Fujian, Fuzhou	26°5'8"N 119°17'35"E	OM569527	OM569588
Lemna gibba				
DW102	Ukraine, Donetsk Oblast, Mironovsky	48°28'04"N 38°16'14"E	OM569528	OM569589
Lemna minor				
DW31	Ukraine, Kyiv Oblast Tacenky	50°09'22"N 30°40'21"E	OM569529	OM569590
DW38	Ukraine, Kyiv Oblast, Maliutianka	50°17'25"N 30°16'20"E	OM569530	OM569591
DW41	Ukraine, Kyiv, Feofaniia	50°20'28"N 30°29'23"E	OM569531	OM569592
DW72	Ukraine, Cherkasy Oblast, Luka, river Ros	49°34'34"N 31°26'51"E	OM793612	OM793617
DW73	Ukraine, Rivne Oblast, Pasiky	50°16'36"N 25°25'58"E	OM793613	OM793618
DW74	Ukraine, Khmelnytskyi Oblast, Kamianets-Podilskyi	48°40'24"N 26°33'57"E	OM793614	OM793619
DW76	Ukraine, Cherkasy Oblast, Korsun-Shevchenkivskyi	49°26'07"N 31°15'04"E	OM793615	OM793620
DW99	Ukraine, Sumy Oblast, Lyntvarivka	50°58'58"N 34°36'57"E	OM569532	OM569593
DW105	Ukraine, Volyn Oblast, Vysochne	51°52'34"N 24°31'08"E	OM569533	OM569594
K38	Ukraine, Kyiv Oblast, Borodyanka, Zdvyzh River	50°38'02"N 29°55'42"E	OM569534	OM569595
K39	Ukraine, Kyiv Oblast, Borodyanka, Zdvyzh River	50°38'02"N 29°55'42"E	OM569535	OM569596
K41	Ukraine, Kyiv Oblast, Borodyanka, Zdvyzh River	50°38'02"N 29°55'42"E	OM569536	OM569597
K43	Ukraine, Kyiv Oblast, Horenka, Irpin River	50°33'19"N 30°17'02"E	OM569537	OM569598
K44	Ukraine, Kyiv Oblast, Horenka, Irpin River	50°33'19"N 30°17'02"E	OM569538	OM569599
K45	Ukraine, Kyiv Oblast, Vorzel	50°33'06"N 30°09'19"E	OM569539	OM569600

K46	Ukraine, Kyiv Oblast, Nemishajeve	50°34'21"N 30°05'26"E	OM569540	OM569601
K48	Ukraine, Kyiv Oblast, Nemishajeve	50°34'21"N 30°05'26"E	OM569541	OM569602
Ya2	China, Shaanxi, Yangling	34°18'06"N 108°02'22"E	OM569542	OM569603
Lemna trisulca				
DW34	Ukraine, Kyiv Oblast, Kruhlyk	50°18'12"N 30°26'35"E	OM569543	OM569604
DW95	Ukraine, Gluboke Lake (The Chernobyl Nuclear Power Plant Exclusion Zone)	51°26'39" N 30°03'54" E	OM569544	OM569605
DW97	Ukraine, Pripyat river (The Chernobyl Nuclear Power Plant Exclusion Zone)	51°16'36" N 30°14'28" E	OM569545	OM569606
K49	Ukraine, Kyiv Oblast, Nemishajeve	50°34'21"N 30°05'26"E	OM569546	OM569607
Lemna turionifera				
DW101	Ukraine, Donetsk Oblast, Mironovsky	48°28'04"N 38°16'14"E	OM569547	OM569608
NB0013	China, Jiangsu, Huai’an	33°37'07"N 119°00'07"E	MZ436179	MZ436180
P2	China, Jiangsu, Huai’an	33°35'52"N 119°03'09"E	OM569548	OM569609
Wolffia arrhiza				
DW32	Ukraine, Kyiv Oblast Tacenky	50°09'22"N 30°40'21"E	OM569549	OM569610
DW35	Ukraine, Kyiv Oblast, Kruhlyk	50°18'12.5"N 30°26'35.9"E	OM569550	OM569611
Wolffia globosa				
C2	China, Jiangsu, Huai’an	33°34'39"N 118°51'30"E	OM569551	OM569612
NB0015	China, Jiangsu, Huai’an	33°33'24"N 118°51'38"E	MZ436183	MZ436184
